# A mechanistic quantitative systems pharmacology framework for glucosamine sulfate in knee osteoarthritis: linking exposure, cartilage biology, and clinical outcomes

**DOI:** 10.3389/jpps.2026.16471

**Published:** 2026-10-09

**Authors:** Marzieh Soheili, Hamed Gilzad Kohan

**Affiliations:** College of Pharmacy, Western New England University, Springfield, MA, United States

**Keywords:** bioavailability, disease-modifying osteoarthritis drug, glucosamine, osteoarthritis, quantitative systems pharmacology

## Abstract

**Background:**

Glucosamine is widely used for osteoarthritis management, yet clinical trial results remain controversial, despite robust preclinical anti-inflammatory effects that fail to translate consistently into clinical benefit. We hypothesized that a mechanistic QSP framework could reconcile these contradictory findings by quantifying how differences in drug exposure at the target site translate into variable treatment effects.

**Objective:**

To develop and validate a quantitative systems pharmacology (QSP) model that mechanistically integrates glucosamine pharmacokinetics, cartilage matrix dynamics, and clinical outcomes in knee osteoarthritis, and to identify pharmacokinetic determinants of clinical efficacy.

**Methods:**

A systematic literature review identified 177 records, from which five landmark studies were selected (three for calibration, one for external validation, and one for exploratory comparison). The QSP model comprises three modules: pharmacokinetics (steady-state synovial exposure), cartilage matrix dynamics (GAG turnover with anti-catabolic drug effect), and clinical outcomes (joint space width [JSW], WOMAC pain). The model implements a primarily anti-catabolic mechanism with minor synthesis stimulation (E_max,syn_ = 0.15). Five parameters were estimated using multi-start optimization against 3-year JSW data from Reginster et al. and Pavelka et al., and 24-week pain data from GAIT (2006). External validation used the GUIDE 2007 trial. Virtual population simulations (N = 500) characterized interindividual variability, and translational simulations explored dose-response, bioavailability enhancement, treatment duration, and patient stratification.

**Results:**

The model was calibrated to reproduce the 3-year JSW treatment effect (0.211 mm predicted vs. 0.240 mm observed; 12% error), a constrained fit conditional on the assumed anti-catabolic potency. The anti-catabolic effect parameter (I_max,deg_) was estimated at its upper bound of 1.00. However, a wide-range re-calibration analysis showed that this predicted benefit is contingent on the assumed anti-catabolic potency: when IC_50,deg_ was varied across the range at which glucosamine’s effects are actually observed *in vitro* (≈10–1,000 μg/mL), the predicted structural benefit fell below the minimal clinically important difference and the model fit degraded substantially. External validation against GUIDE 2007 reproduced both arms’ pain trajectories (mean absolute error 3.8 WOMAC points; all observations within the 90% prediction intervals) but under-predicted the between-arm treatment effect (−1.4 vs. −5.0 WOMAC points), indicating the model captures overall pain magnitude better than the glucosamine-attributable difference; systematic overprediction of pain reduction further suggests population-specific heterogeneity in placebo response. Under the optimistic potency anchor, translational simulations predicted a minimum effective dose of approximately 750 mg/day, that 83% of patients achieve the 0.1 mm MCID at the standard dose under real-world variability (98.2% show positive benefit, treatment effect >0, under calibration conditions), 73% efficacy enhancement through bioavailability improvement from 22% to 40%, continued structural benefit through 5 years without plateau, and consistent treatment effects (NNT = 2) across disease severity stages. All of these predictions are contingent on the same anti-catabolic potency assumption; under in vitro–consistent potency, the predicted benefit falls below clinical significance (see Conclusions and “Re-calibration across the biologically realistic potency range”).

**Conclusion:**

This QSP model provides a mechanistic framework for examining the conditions under which glucosamine could exert disease-modifying effects in knee osteoarthritis. Critically, the model’s predicted structural benefit is contingent on optimistic assumptions about both anti-catabolic potency and oral bioavailability; under values consistent with the available *in vitro* and pharmacokinetic evidence, the predicted benefit at achievable joint concentrations is minimal. This dependence offers a mechanistic explanation for the inconsistent and frequently null results of independent glucosamine trials. However, the assumption that salt form determines bioavailability is challenged by recent pharmacokinetic and crystallographic evidence suggesting that commercially available glucosamine sulfate formulations may not differ fundamentally from glucosamine hydrochloride in their active moiety. Model predictions are contingent on the calibration data from industry-sponsored European trials, and should be interpreted in light of the failure of independent trials to replicate these structural benefits. This work exemplifies how Model-Informed Drug Development approaches can quantify the sensitivity of clinical outcomes to pharmacokinetic parameters, informing future trial design and dose optimization.

## Introduction

### Global burden of osteoarthritis

Osteoarthritis (OA) is the most prevalent form of arthritis and a leading cause of musculoskeletal disability worldwide, affecting approximately 595 million people globally [1–3]. The knee is the most commonly affected joint, accounting for over 56% of cases [3, 4]. Current management remains primarily palliative, and no disease-modifying osteoarthritis drug (DMOAD) has received FDA or EMA approval [5]. This substantial unmet need, compounded by projections of 75%–95% increases in prevalence by 2050 [1, 3], underscores the urgency of developing mechanistic frameworks to guide therapeutic optimization.

### The unmet need for disease-modifying osteoarthritis drugs (DMOADs)

Despite decades of research, multiple mechanistically promising DMOAD candidates have failed to achieve consistent structural modification in clinical trials [6–8]. Key explanations include OA heterogeneity across molecular endotypes [9–11], the insensitivity of radiographic joint-space width (JSW) as a structural endpoint [12, 13], and the historical treatment of OA as a homogeneous disease in clinical trial design [10, 11]. These challenges argue for mechanistic, systems-level approaches such as Quantitative Systems Pharmacology (QSP) to integrate biology, pharmacokinetics, and clinical endpoints.

### Glucosamine: pharmacology and mechanisms of action

Glucosamine, administered as glucosamine sulfate or glucosamine hydrochloride, is widely used for OA management [14, 15]. As an amino monosaccharide, it serves as an essential substrate for glycosaminoglycan (GAG) biosynthesis [16, 17]. Pharmacologically, glucosamine primarily exerts anti-catabolic effects by inhibiting IL-1β–induced inflammatory signaling, suppressing NF-κB activation, and reducing the expression of matrix metalloproteinases (MMP-1, MMP-3, MMP-13) and pro-inflammatory mediators (COX-2, PGE2, TNF-α) in human osteoarthritic chondrocytes [18–20]. Importantly, glucosamine does not reliably enhance anabolic matrix synthesis at therapeutic concentrations; comparative studies confirm that its chondroprotective effects derive primarily from limiting cytokine-driven matrix degradation rather than stimulating new matrix production [21–23].

### Pharmacokinetics and bioavailability

Glucosamine exhibits rapid absorption, extensive first-pass metabolism, and low oral bioavailability (estimated at ∼19% from preclinical data; absolute human bioavailability has not been determined owing to the absence of an intravenous reference [24–26]), attributed primarily to gut-wall presystemic loss rather than hepatic extraction. Absorption is primarily mediated by facilitated transport through glucose transporters (GLUTs) rather than by passive diffusion, indicating that membrane permeability is not the rate-limiting step in oral absorption [24, 25]. Following a 1,500 mg once-daily dose, plasma concentrations increase ∼30-fold from baseline, reaching a Cmax of ∼10 μM at 3 h [26]. Glucosamine is subsequently eliminated in a multi-exponential fashion with substantial distribution into extravascular compartments; plasma concentrations remain measurable and above baseline for up to 48 h, and the terminal half-life was tentatively estimated at ∼15 h [26]. This sustained systemic exposure, together with synovial fluid concentrations that remain elevated at 12 h post-dose [27], supports once-daily dosing and the use of a steady-state synovial concentration as the pharmacodynamic driver (developed further in Module A: Pharmacokinetics). The glucosamine controversy has been characterized as fundamentally a pharmacokinetic issue [28], with preclinical studies demonstrating dose/concentration-dependent anti-inflammatory effects in adjuvant arthritis models [29, 30]. However, the assumption that crystalline glucosamine sulfate (cGS) has superior bioavailability compared with other glucosamine salt forms has recently been challenged. A randomized, double-blind, crossover pharmacokinetic study comparing cGS with regular glucosamine sulfate (rGS) in healthy volunteers found no significant difference in AUC_0–24_, Cmax, or Tmax between formulations, with rGS showing numerically higher systemic exposure (AUC_0–24_ GMR = 1.69, favoring rGS, p = 0.136) [31].

Furthermore, crystallographic analyses have demonstrated that commercially available “glucosamine sulfate” products, including stabilized “crystalline” forms, are physical mixtures of glucosamine chloride and potassium sulfate (or sodium chloride) rather than true sulfate salts [32]. Genuine glucosamine sulfate is highly hygroscopic and chemically unstable, precluding formulation into stable commercial dosage forms [32]. Since glucosamine sulfate dissociates in gastric fluid to yield the same glucosamine cation as glucosamine hydrochloride, the pharmacologically active moiety is identical regardless of the starting salt form [31, 32]. The USP reference standard for glucosamine is based on the hydrochloride salt, further underscoring that distinctions between commercial “sulfate” and “hydrochloride” products may reflect differences in excipients, manufacturing quality control, and labeling rather than fundamental differences in the active ingredient [33].

Despite these formulation controversies, glucosamine achieves therapeutically relevant synovial concentrations, with selective cartilage accumulation and prolonged synovial retention relative to plasma [27, 34–36]. This biphasic profile, with rapid initial plasma distribution but sustained synovial exposure, motivates the use of synovial fluid concentration as the driver of cartilage effects in the QSP model; the supporting data are detailed in the Methods (Module A: Pharmacokinetics).

### Clinical evidence and the glucosamine controversy

The clinical efficacy of glucosamine remains contentious. Two pivotal European trials, both funded by the manufacturer of a patented crystalline glucosamine sulfate product, demonstrated significant structure-modifying effects over 3 years, with minimal joint space narrowing (−0.06 mm) compared with placebo (−0.19 to −0.31 mm), as well as symptomatic improvement [37, 38]. These structural benefits have not been replicated in independent trials. In contrast, the NIH-funded GAIT trial using glucosamine hydrochloride found no significant overall benefit over placebo, though patients with moderate-to-severe pain showed benefit with combination therapy (glucosamine hydrochloride 1,500 mg/day plus chondroitin sulfate 1,200 mg/day) [39]. Notably, 78% of GAIT participants had mild baseline pain, and the placebo response rate was 60.1%, substantially higher than typically observed in OA trials enrolling more symptomatic patients [39]. The controversy has historically been attributed to differences in formulation [28], but recent evidence challenges this interpretation. Pharmacokinetic comparisons have found no significant bioavailability advantage for crystalline glucosamine sulfate over regular glucosamine sulfate [31], and crystallographic evidence suggests both products deliver the same active moiety (glucosamine chloride) after gastric dissolution [32]. Therefore, the inconsistent trial results likely reflect a combination of factors: (1) differences in trial design, patient selection, and outcome assessment methodology; (2) the absence of dose-optimization studies, as every trial has used the same 1,500 mg dose established decades ago, without evidence of optimality [40]; (3) heterogeneity in patient populations and placebo response rates [41]; and (4) the reliance of positive structural outcomes on industry-sponsored trials that have not been independently replicated. A further consideration is that the glucosamine content of commercial formulations has been shown to vary substantially from label claims [42], and the trials reviewed here administered products based on labeled doses without independent verification of actual potency. The nominal doses reported across studies, including those tabulated in Table 1, therefore reflect label claims rather than confirmed delivered potency, introducing an additional source of variability that is independent of formulation bioavailability.

**TABLE 1:** Core calibration studies.

Study	N	Formulation	Dose	Duration	Primary outcome	Use in model
Reginster 2001 [38]	212	Crystalline GS	1,500 mg/day	3 years	JSW change	Calibration (JSW)
Pavelka 2002 [37]	202	Crystalline GS	1,500 mg/day	3 years	JSW change	Calibration (JSW)
GAIT 2006 [39]	1,583	GH	1,500 mg/day	24 weeks	WOMAC pain	Calibration (pain)
GUIDE 2007 [51]	318	Crystalline GS	1,500 mg/day	6 months	WOMAC pain	External validation (pain)
MOVES 2015 [52]	606	GH + CS (combination)	GH 1500 + CS 1200 mg/day	6 months	WOMAC pain	Exploratory comparison

GS, glucosamine sulfate; GH, glucosamine hydrochloride; CS, chondroitin sulfate; JSW, joint space width; WOMAC, Western Ontario and McMaster Universities Osteoarthritis Index; MOVES, Multicentre Osteoarthritis interVEntion trial with Sysadoa. MOVES N = 606 reflects total randomized participants; the 264 and 258 reported in Exploratory Model Comparison are the two analyzed treatment arms.

### Implications for QSP modeling

The glucosamine controversy presents an ideal case study demonstrating where traditional empirical approaches fall short and mechanistic modeling becomes essential. The disconnect between robust preclinical anti-inflammatory effects and inconsistent clinical outcomes suggests that simple dose-response relationships cannot capture the complexity of glucosamine’s action in OA. Although empirical pharmacokinetic and pharmacodynamic approaches are developed to adequately describe the data, they are generally not designed to provide quantitative insights into specific underlying mechanisms or to facilitate the use of these mechanistic insights to extrapolate to new conditions [43].

Key modeling implications include: (1) the need to integrate pharmacokinetics (absorption variability, synovial distribution) with pharmacodynamics (IL-1β/NF-κB pathway inhibition, cartilage turnover); (2) accounting for disease heterogeneity and patient stratification based on OA severity; (3) linking molecular-level mechanisms to clinically measurable endpoints such as WOMAC scores and joint space width; and (4) exploring dose optimization beyond the empirically established 1,500 mg daily regimen. A QSP framework can reconcile the apparent contradiction between mechanistic evidence and clinical trial results by explicitly modeling the conditions under which therapeutic concentrations are achieved at target tissues.

### Rationale for QSP modeling

The disconnect between robust preclinical anti-inflammatory effects and inconsistent clinical outcomes suggests that empirical dose-response relationships cannot capture the complexity of glucosamine’s action. Quantitative Systems Pharmacology (QSP) integrates computational modeling across biological and temporal scales and species [44], making it uniquely suited to address this challenge. Glucosamine’s mechanism spans absorption kinetics, plasma-synovial fluid partitioning, intracellular uptake, and downstream NF-κB inhibition, affecting cartilage homeostasis; processes that require mechanistic integration rather than empirical fitting.

The regulatory landscape increasingly supports such approaches. The FDA Modernization Act 2.0 (December 2022) removed the blanket mandate for animal testing, allowing computational and other non-animal methods as alternatives in drug development [45]. QSP submissions to the FDA have increased steadily, exceeding 80 submissions in 2023 alone [46].

Despite extensive investigation, many disease-modifying osteoarthritis drug (DMOAD) programs have failed to demonstrate consistent clinical benefit [8, 47]. Increasingly, these failures are recognized not solely as biological shortcomings but as challenges in quantitative translation between drug exposure, tissue pharmacology, and clinical outcomes. Model-Informed Drug Development (MIDD) frameworks integrate pharmacokinetics, pharmacodynamics, and systems biology to reduce uncertainty in dose selection and clinical trial design [48, 49]. Within this context, QSP models provide a mechanistic framework that links drug exposure to disease progression, enabling hypothesis testing, evaluation of variability drivers, and simulation of clinical scenarios prior to costly trials [43, 50].

### Objectives

Despite extensive literature, no comprehensive QSP model integrates glucosamine pharmacokinetics, mechanisms, and clinical outcomes in OA. Such a model would: (1) quantitatively reconcile contradictory trial results through a mechanistic understanding of PK differences; (2) predict optimal dosing strategies and formulations; (3) identify patient populations most likely to benefit; and (4) exemplify model-informed drug development (MIDD) aligned with the FDA Modernization Act 2.0 principles.

Glucosamine is an ideal QSP candidate given extensive human trial data on 3-year structural outcomes; well-characterized pharmacokinetics, including synovial fluid concentrations; elucidated mechanisms (NF-κB inhibition); and validated endpoints (joint space width, WOMAC) with established MCIDs.

The objectives of this study were to: (1) develop a QSP model integrating glucosamine PK, cartilage homeostasis, and clinical outcomes; (2) calibrate against landmark European trial data; (3) validate using independent datasets; and (4) apply translational simulations exploring dose-response, bioavailability enhancement, treatment duration, and patient stratification by disease severity. Beyond characterizing glucosamine pharmacology, this work evaluates whether a mechanistic QSP framework can inform development-relevant questions, including exposure requirements for structural modification, variability in clinical response, and optimization of dosing and formulation strategies.

## Methods

### Literature search and study selection

#### Search strategy

A systematic literature search was conducted in PubMed through December 2024 to identify randomized controlled trials (RCTs) of glucosamine for the treatment of osteoarthritis. A comprehensive primary search strategy was employed to capture all relevant glucosamine RCTs, with targeted subset searches to identify studies of particular interest for model parameterization (Table 2). The primary search retrieved all glucosamine osteoarthritis RCTs regardless of formulation or co-treatment. Within this pool, subset searches flagged studies involving combination therapy with chondroitin sulfate (relevant for assessing potential synergistic effects) and studies reporting cartilage degradation biomarkers (relevant for mechanistic model calibration). All subset results were contained within the primary search, confirming comprehensive coverage.

**TABLE 2:** PubMed search strategies and results.

Strategy	Search terms	Results
Primary search	(“Glucosamine”[MeSH] OR “glucosamine”[tiab]) AND (“osteoarthritis”[MeSH] OR “osteoarthritis”[tiab]) AND (“randomized controlled trial”[pt] OR “clinical trial”[pt])	177
Subset: Combination therapy	(“Glucosamine”[tiab] AND “chondroitin”[tiab]) AND (“osteoarthritis”[tiab]) AND (“randomized”[tiab] OR “placebo”[tiab])	47
Subset: Biomarker outcomes	(“Glucosamine”[tiab]) AND (“CTX-II” OR “COMP” OR “cartilage oligomeric” OR “collagen”[tiab]) AND (“osteoarthritis”[tiab])	19
Total unique records	​	177

#### Study selection and PRISMA flow

Study selection followed the Preferred Reporting Items for Systematic Reviews and Meta-Analyses (PRISMA) guidelines. The primary search yielded 177 records. Within this pool, subset searches identified 47 studies on combination therapy with chondroitin and 19 studies reporting cartilage biomarker outcomes, both of which were flagged for priority review. Title and abstract screening were performed by two independent reviewers against predefined eligibility criteria.

Inclusion criteria: (1) randomized controlled trial design; (2) adult patients with radiographically confirmed osteoarthritis (Kellgren-Lawrence grade ≥2); (3) glucosamine intervention (any salt form, any dose); (4) placebo or active comparator arm; (5) reporting of at least one outcome relevant to model calibration (WOMAC pain, joint space width, or cartilage biomarkers).

Exclusion criteria: (1) non-randomized or uncontrolled study designs; (2) preclinical or *in vitro* studies; (3) interventions not including glucosamine as the primary component; (4) pediatric populations; (5) conference abstracts without full methodology.

Screening excluded 32 records: 18 non-controlled trials or observational studies, seven studies in which glucosamine was not the primary intervention, 4 preclinical or *in vitro* studies, and three conference abstracts without full methodology. This yielded 145 studies meeting eligibility criteria for potential model parameterization.

#### Priority study selection

From the 145 eligible studies, a tiered prioritization framework was applied to select studies providing the highest-quality data for model calibration and validation. Priority was determined by: (1) formulation type, with crystalline glucosamine sulfate receiving highest priority due to the availability of long-term structural outcome data from European trials; (2) outcome type, with structural outcomes (joint space width) prioritized for disease-modifying assessment, followed by biomarkers for mechanistic validation, and clinical outcomes (WOMAC) for symptomatic efficacy; (3) study duration, with longer-duration trials (≥2 years) preferred for structural outcomes; (4) methodological quality based on randomization, blinding, and sample size (Table 3).

**TABLE 3:** Priority tier classification of selected studies.

Priority tier	Criteria	Studies (n)
Tier 1 (highest)	Crystalline GS, JSW outcome, ≥2 years, RCT	8
Tier 2	Any GS formulation, JSW, or biomarker outcome, ≥1 year	23
Tier 3	GS or GH, WOMAC outcome, ≥12 weeks	32
Tier 4 (lowest)	Any glucosamine, any OA outcome	82
Total eligible studies	​	145

GS, glucosamine sulfate; GH, glucosamine hydrochloride; JSW, joint space width; RCT, randomized controlled trial.

#### Full-text retrieval and accessibility

Full-text retrieval was attempted for all 63 priority studies. Automated downloads from PubMed Central and publisher open-access repositories successfully retrieved 32 PDFs. The remaining 31 studies were not freely accessible due to: (1) publication in non-English language journals without English full-text (primarily German, Italian, and Czech publications from the 1990s–2000s); (2) paywall restrictions without institutional access; (3) journals no longer in publication or with incomplete digital archives. Text extraction using the pdftools R package successfully processed 80 of 81 available PDFs (including supplementary materials), yielding 725 pages of extracted text to support systematic data extraction. One PDF (PMID 9001835) failed extraction due to file corruption.

Final calibration dataset: Five landmark studies with complete outcome reporting were selected as the core calibration dataset (Table 1). These studies were selected based on: comprehensive longitudinal data, standardized outcome measures, well-characterized patient populations, and historical significance in establishing the glucosamine evidence base.

### Data extraction and quality assurance

A structured data extraction template was developed in Microsoft Excel with separate worksheets for: study characteristics (design, population, intervention), WOMAC pain outcomes (mean, SD, change from baseline by timepoint), joint space width measurements (absolute values, change, measurement method), cartilage biomarkers (CTX-II, COMP concentrations), pharmacokinetic parameters (absorption rate, bioavailability, clearance), placebo response dynamics, and responder rates. The template included standardized fields for data source documentation (table number, figure, text page) and quality flags.

Data extraction was performed independently by two reviewers using the extracted PDF text files. Discrepancies were resolved by consensus or reference to original publications. For outcomes reported only in figures, data were extracted using WebPlotDigitizer with inter-rater reliability assessment. When standard deviations were not reported, they were estimated from standard errors, 95% confidence intervals, or imputed from studies with similar populations following Cochrane Handbook recommendations.

Quality assurance included: (1) verification of extracted values against original publications; (2) unit standardization (WOMAC scores normalized to 0–100 scale, JSW in mm); (3) documentation of scale transformations; (4) flagging of imputed or estimated values. All extraction discrepancies and corrections were documented in the verification log.

#### Risk of bias assessment

The five core studies used for model calibration and validation were assessed for risk of bias using the Cochrane Risk of Bias 2 (RoB 2) tool for randomized trials. Each study was evaluated across five domains: randomization process, deviations from intended interventions, missing outcome data, measurement of the outcome, and selection of the reported result. Risk of bias judgments (low, some concerns, high) were made independently by two reviewers, with disagreements resolved by consensus. The complete risk of bias assessment is presented in Supplementary Table S7.

### QSP model structure

The quantitative systems pharmacology model integrates three interconnected modules representing glucosamine pharmacokinetics, cartilage matrix dynamics, and clinical outcomes. The model was implemented as a system of ordinary differential equations that explicitly represents the mechanistic pathway from drug exposure to clinical benefit. The complete set of model equations and parameter details is provided in the Supplementary Appendix.

#### Module A: pharmacokinetics

Glucosamine pharmacokinetics were modeled using a steady-state exposure approach. Given the once-daily dosing regimen and the focus on chronic treatment outcomes over months to years, steady-state average synovial concentrations were used as the pharmacodynamic driver:

$$C_{\text{syn},\text{ss}}=R_{\text{syn}:\text{plasma}}\;\cdot \;\frac{F\;\cdot \;\text{Dose}}{\text{CL}\;\cdot \;\tau }$$

where F is oral bioavailability, Dose is the administered dose (typically 1,500 mg), CL is apparent clearance (12 L/h), τ is the dosing interval (24 h), and R_syn:plasma_ is the synovial-to-plasma concentration ratio. Because bioavailability and clearance enter this expression only through the ratio F/CL, the steady-state synovial concentration, and therefore the predicted treatment effect, depends on systemic exposure rather than on F or CL individually. Uncertainty in clearance is thus mathematically equivalent to a proportional change in bioavailability and is captured by the bioavailability sensitivity analysis (F = 10%–40%; Supplementary Figure S4). For the base case, F values of 0.22 for glucosamine sulfate and 0.11 for glucosamine hydrochloride were adopted from published pharmacokinetic studies [26]. However, recent evidence challenges the assumption of a two-fold difference in bioavailability between these formulations [31, 32]; sensitivity analyses examining the impact of equalizing bioavailability values are presented in the Discussion.

Justification for using average steady-state synovial concentration rather than dynamic plasma exposure: Although glucosamine plasma concentrations fluctuate within the once-daily dosing interval, several considerations support the use of steady-state average synovial concentrations rather than dynamic plasma profiles for modeling chronic cartilage effects. First, glucosamine displays a relatively long, tentatively estimated terminal half-life (∼15 h) with plasma concentrations sustained above baseline for up to 48 h [26], and synovial fluid concentrations remain elevated at 12 h post-dose [27] so the synovial compartment is exposed to drug throughout the dosing interval rather than only transiently. Second, although steady-state synovial glucosamine concentrations are somewhat lower than simultaneous plasma concentrations (median 4.34 μM vs. 7.17 μM at 3 h post-dose) [35], synovial exposure is sustained and the joint maintains therapeutically relevant concentrations, supporting the use of a synovial compartment as the pharmacodynamic driver [34, 35]. Third, the pharmacodynamic endpoint (cartilage GAG turnover) operates on a timescale of weeks to months, rendering hour-to-hour plasma fluctuations less relevant than sustained average exposure. Fourth, Persiani et al. [35] reported a median synovial fluid glucosamine concentration of 4.34 μM in osteoarthritic patients at steady state, sampled 3 h after the last of 14 once-daily 1,500 mg doses. This 4.34 μM value corresponds to ∼0.78 μg/mL, and the measured median synovial:plasma concentration ratio in that study was ∼0.76 [35] (median of individual patient ratios; the ratio of the median concentrations, 4.34/7.17 µM, is ∼0.61). For computational efficiency, the steady-state synovial concentration was implemented using an equivalent reference-scaling approach (see Module A: Pharmacokinetics), in which standard dosing (F_sulfate_ = 0.22, Dose = 1,500 mg) yields an effective reference concentration of approximately 0.30 μg/mL at the target site, with other formulations and doses scaled proportionally. This reference is deliberately conservative: it lies below the measured 3-h steady-state synovial concentration (∼0.78 μg/mL [35]) and approximates a dosing-interval average rather than a peak. Because a lower assumed target-site concentration reduces, rather than inflates, the predicted drug effect, this choice does not favour a positive efficacy result; the sensitivity of predictions to higher target-site exposure is bracketed by the bioavailability analysis (F = 10–40%; Supplementary Figure S4), which spans the measured synovial exposure.

A two-fold difference in bioavailability between glucosamine sulfate and glucosamine hydrochloride formulations was assumed; we note that this assumption is not firmly established, as recent evidence questions whether any such difference reflects true formulation-dependent pharmacokinetics or methodological differences between studies [31, 32]. The model’s sensitivity to this parameter is explicitly examined in the translational simulations (Supplementary Figure S4). The IC_50_ for degradation inhibition was set to a nominal value of 3.0 μg/mL (16.7 μM, based on the glucosamine free-base molecular weight of 179.17 g/mol). We note that this value is not a measured IC_50_ from a specific study: although glucosamine sulfate inhibits IL-1β–induced NF-κB activation and COX-2 expression in human chondrocytes [53], that inhibition was observed over a concentration range of approximately 10–1,000 μg/mL, and no formal IC_50_ was reported. The 3.0 μg/mL value should therefore be regarded as an assumed potency anchor rather than an experimentally determined constant, and its influence on model predictions is examined extensively (Model Calibration Results; Supplementary Table S4; and the wide-range re-calibration analysis in “Re-calibration across the biologically realistic potency range”, Supplementary Figure S6 and Supplementary Table S8).

#### Module B: cartilage matrix dynamics

Cartilage homeostasis was represented by a GAG (glycosaminoglycan) turnover model where glucosamine acts through a primary anti-catabolic mechanism supplemented by minor synthesis stimulation:

$$\frac{d\text{GAG}}{dt}=k_{\text{syn}}\;\cdot \;E_{\text{syn}}-k_{\deg}\;\cdot \;\left(1-\frac{I_{\max,\deg}\;\cdot \;C_{\text{syn}}}{IC_{50,\deg}+C_{\text{syn}}}\right)\;\cdot \text{GAG}$$

Where: $E_{\text{syn}}=1+\frac{E_{\max,\text{syn}}\;\cdot \;C_{\text{syn}}}{EC_{50,\text{syn}}+C_{\text{syn}}}$

A minor synthesis stimulation term was included based on *in vitro* evidence that glucosamine sulfate stimulates proteoglycan production in human osteoarthritic chondrocytes [54]. We emphasize, however, that the glucosamine concentrations required to stimulate proteoglycan synthesis in cell and cartilage studies are generally 10 to 1000-fold higher than the transient concentrations achieved in serum or synovial fluid after oral ingestion [55], the same concentration mismatch that applies to the anti-catabolic term. Because the stimulatory effect was observed at supraphysiological concentrations (10–100 μg/mL), the synthesis-stimulation parameters were assigned conservative nominal values rather than fitted to the *in vitro* data (Emax,syn = 0.15, a maximum 15% increase in synthesis rate; EC50,syn = 2.0 μg/mL), keeping the anabolic component minor relative to the primary anti-catabolic mechanism. Like the anti-catabolic potency, this anabolic term should therefore be regarded as a nominal assumption whose physiological relevance at achievable exposures is uncertain. This effect is substantially smaller than that of the primary anti-catabolic component: at the reference synovial concentration (0.30 μg/mL), degradation inhibition results in approximately a 9% reduction in degradation rate, while synthesis stimulation results in approximately a 2% increase in synthesis rate.

#### Module C: clinical outcomes

##### Joint space width

Joint space width (JSW) was linked to GAG content through a power relationship reflecting the contribution of cartilage matrix to radiographic joint space:

$$\text{JSW}\left(t\right)={JSW}_{0}\;\cdot \;{\left(\frac{\text{GAG}\left(t\right)}{{GAG}_{0}}\right)}^{\gamma }$$

where JSW_0_ is baseline joint space width (fixed at 4.2 mm, the approximate mean baseline values reported in the calibration trials [37, 38]), GAG_0_ is baseline GAG content, and γ is a shape parameter (fixed at 1.0), representing a linear relationship between normalized GAG content and radiographic JSW. This simplifying assumption was adopted because sufficient data were not available to identify a nonlinear exponent; future models incorporating MRI-based cartilage composition data could estimate γ directly.

##### WOMAC pain

Pain was modeled as the sum of four components representing baseline disease severity, structural pain from cartilage degradation, placebo response, and direct drug-mediated symptomatic relief:

$$\text{Pain}\left(t\right)={Pain}_{0}+\alpha_{\text{struct}}\;\cdot \;\max\left(0,{GAG}_{0}-\text{GAG}\left(t\right)\right)-P_{\max}\;\cdot \;\left(1-e^{-k\_pl\;\cdot \;t}\right)-\frac{\beta_{\text{drug}}\;\cdot \;C_{\text{syn}}}{EC_{50,\text{pain}}+C_{\text{syn}}}$$

where *Pain*_0_ is the baseline WOMAC pain score (estimated parameter), *α*_struct_ is the pain sensitivity to cartilage loss (fixed at 150 WOMAC points per unit GAG, based on the scaling that complete GAG loss from baseline would correspond to substantial pain increase), *GAG*_0_ *− GAG*(*t*) represents cumulative cartilage loss from baseline, *P*_max_ is the maximum placebo effect (estimated parameter), *k*_pl_ is the placebo onset rate constant (fixed at 0.02 days^−1^), *β*_drug_ is the maximum direct analgesic effect (fixed at 5.0 WOMAC points), and *EC*_50,pain_ is the concentration for half-maximal direct effect (fixed at 3.0 μg/mL). At the reference synovial concentration (0.30 μg/mL), the direct analgesic component contributes only 0.45 WOMAC points, confirming that glucosamine’s pain-relieving effects are primarily mediated through structural preservation rather than direct analgesia.

##### Mechanistic assumptions

The linkage of pain reduction to GAG preservation represents a simplifying assumption rather than a claim of direct causation. OA pain is multifactorial, involving synovitis, bone marrow lesions, central sensitization, and psychosocial factors, all of which are only partially correlated with cartilage status. The GAG-pain pathway in this model should be interpreted as a phenomenological representation that captures the observation that interventions that preserve cartilage structure tend to provide symptomatic benefit over time, without implying that GAG content directly determines nociceptive signaling. This formulation is consistent with the delayed onset of glucosamine’s symptomatic effects (weeks to months) and the correlation between structural and symptomatic outcomes observed in the calibration trials. Consequently, nearly all pain reduction in the glucosamine arm relative to placebo derives from the GAG preservation pathway, with a minor contribution (approximately 0.45 WOMAC points) from the direct analgesic component. All WOMAC pain scores are expressed on the normalized 0–100 scale, where 0 represents no pain, and 100 represents maximum pain. We further note that, because the symptomatic benefit in this model derives predominantly from the structural (GAG preservation) pathway, the physiological relevance of the pain predictions is itself contingent on the anti-catabolic potency assumption examined in “Re-calibration across the biologically realistic potency range”; if glucosamine’s structural effect is negligible at achievable synovial concentrations, the model’s predicted symptomatic benefit would be correspondingly limited.

#### Module D: biomarker dynamics

The model structure includes provisions for cartilage degradation biomarkers (CTX-II, COMP) as indirect response markers linked to the degradation process. However, biomarker dynamics were not calibrated in the current analysis due to limited longitudinal biomarker data available from the selected trials. Future model extensions incorporating biomarker endpoints could provide additional mechanistic validation independent of clinical outcomes. The complete model structure integrating all three modules is illustrated in Figure 1.

**FIGURE 1:**
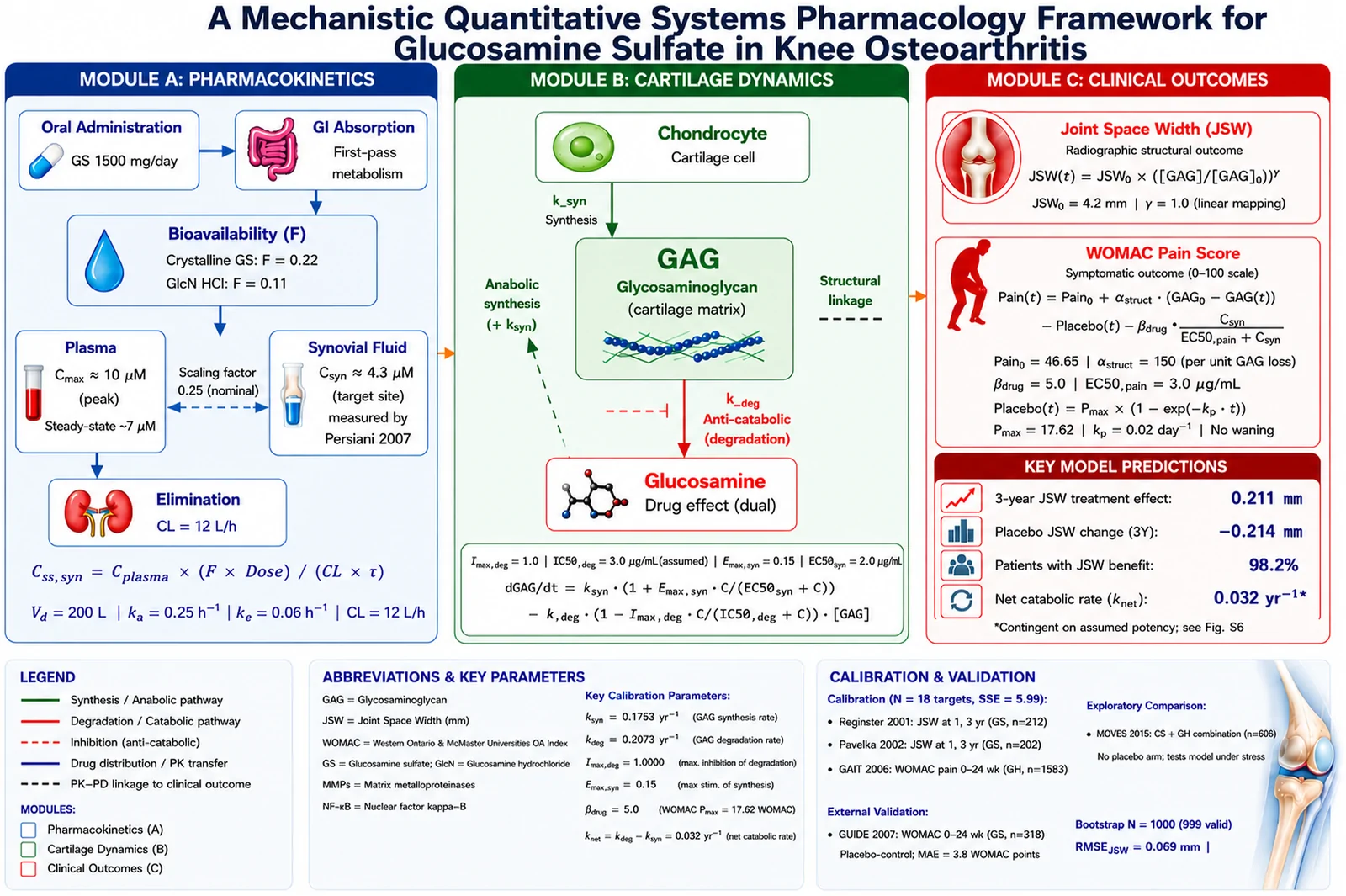
Schematic of a quantitative systems pharmacology model for glucosamine sulfate in knee osteoarthritis. The model comprises three interconnected modules: **(A)** Pharmacokinetics; oral absorption, bioavailability, and synovial fluid distribution; **(B)** Cartilage Matrix Dynamics; GAG turnover with glucosamine-mediated inhibition of the IL-1β/NF-κB/MMP degradation cascade (primarily anti-catabolic mechanism with minor synthesis stimulation (E_max,syn_ = 0.15)); and **(C)** Clinical Outcomes; joint space width (structural endpoint) and WOMAC pain score (symptomatic endpoint) linked to GAG content. Key model predictions, parameter values, and calibration/validation data sources are shown in the bottom panels. Abbreviations are defined in the figure.

### Model calibration

Five parameters were estimated by weighted least-squares: cartilage turnover rates (k_syn_, k_deg_), glucosamine anti-catabolic effect (I_max,deg_), and pain dynamics (Pain_0_, P_max_). Pharmacokinetic parameters (Fsulfate = 0.22, FHCl = 0.11, CL = 12 L/h, Ceff,ref = 0.30 μg/mL) and the potency anchor (IC50,deg = 3.0 μg/mL) were fixed rather than estimated. We note that absolute oral bioavailability and systemic clearance have not been directly determined for glucosamine in humans; Persiani et al. [26] characterized plasma pharmacokinetics but could not estimate absolute bioavailability (no intravenous arm), and the available F estimates derive from animal studies (≈3–6% in horses, 12% in dogs, and 21% in rats [24], using glucosamine hydrochloride). The values adopted here should therefore be regarded as plausible assumed inputs. Because F and CL enter the steady-state exposure only through the ratio F/CL, uncertainty in clearance is equivalent to a proportional change in bioavailability and is captured by the bioavailability sensitivity analysis (Supplementary Figure S4). We further note that the published steady-state plasma AUC over the dosing interval (∼14,560 ng·h/mL at 1,500 mg [26]) implies an apparent oral clearance (CL/F) of ∼100 L/h; the nominal CL and F adopted here are therefore illustrative, literature-informed inputs rather than independently fitted quantities. The target-site driver in the implemented model is fixed by the effective synovial reference (∼0.30 μg/mL, Module A: Pharmacokinetics), which is set conservatively relative to the measured synovial data [35] and does not depend on the individual values of CL or F; the associated exposure uncertainty is bracketed by Supplementary Figure S4.

Observation Model: An explicit statistical observation model was assumed for each endpoint. For JSW measurements, observations were assumed to follow a normal distribution around model predictions with endpoint-specific measurement error:

$${JSW}_{\text{obs}}\;∼\;N\left({JSW}_{\text{pred}},{\sigma_{\text{JSW}}}^{2}\right)$$

where σ_JSW_ = 0.08 mm, reflecting the standard error of JSW measurement reported in the calibration trials. For WOMAC pain scores:

$${Pain}_{\text{obs}}\;∼\;N\left({Pain}_{\text{pred}},{\sigma_{\text{Pain}}}^{2}\right)$$

where σ_Pain_ = 5.0 WOMAC points, based on typical within-group standard deviations in OA trials. These observation-error estimates were used to compute weighted residuals for diagnostic purposes and to scale the relative contributions of each endpoint to the objective function.

Each optimization enforced box constraints and the structural constraint k_deg_ > k_syn_ (Supplementary Table S6). The resulting calibrated values are specified in the MATLAB pipeline (R2023b) for all downstream simulation, figure generation, and uncertainty analysis; bootstrap confidence intervals and the wide-range re-calibration re-estimate the free parameters using fmincon (interior-point algorithm).

Objective Function: The objective function was the weighted sum of squared errors (WSSE), equivalent to maximum likelihood estimation under the Gaussian observation model:

$$\text{WSSE}=\sum_{i}{\left[\frac{{JSW}_{\text{obs},i}-{JSW}_{\text{pred},i}}{\sigma_{\text{JSW}}}\right]}^{2}+\sum_{j}{\left[\frac{{Pain}_{\text{obs},j}-{Pain}_{\text{pred},j}}{\sigma_{\text{Pain}}}\right]}^{2}$$

This formulation ensures that JSW and WOMAC endpoints contribute to measurement precision in proportion to their precision, avoiding dominance by either endpoint due to scale differences. In the implemented objective, the WOMAC pain residuals were additionally down-weighted relative to the structural endpoint to prioritize the joint-space-width data that drive the disease-modifying predictions. Calibration targets included JSW change at Years 1 and 3 from Reginster 2001 and Pavelka 2002 (n = 8 observations) and WOMAC pain at baseline, Weeks 4, 8, 16, and 24 from GAIT 2006 for both placebo and GH arms (n = 10 observations), yielding 18 observations for estimating 5 parameters.

Data Sparsity Considerations: With 18 observations and 5 estimated parameters, the effective degrees of freedom (df = 13) are modest, limiting the precision of the parameter estimates. This constraint motivated our reliance on literature-derived values for pharmacokinetic and potency parameters rather than attempting to estimate them from the available data. Uncertainty quantification through bootstrap resampling accounts for the influence of individual data points on parameter estimates, and the sensitivity analyses in the Results characterize the robustness of predictions to parameter uncertainty.

Parameter estimates were constrained to physiologically meaningful ranges (E_max_ ≤ 1, I_max_ ≤ 1, k_deg_ > k_syn_). Two structural identifiability considerations merit discussion. First, for inhibitory E_max_ models with fixed potency (IC_50_), only the product I_max_ × (C/IC_50_) is fully identifiable from efficacy data; consequently, uncertainty in IC_50_ propagates inversely to I_max_ estimates. Second, the cartilage turnover parameters k_syn_ and k_deg_ enter the model in a manner that renders only their net balance (k_net_ = k_deg_ − k_syn_) identifiable from JSW progression data; the individual rates are structurally correlated. We therefore report both the individual parameter estimates (for mechanistic interpretability) and the derived net catabolic rate (for inferential validity). The constraint k_deg_ > k_syn_ was imposed based on the biological requirement that OA cartilage exhibits net matrix loss; this assumption is consistent with the progressive JSW narrowing observed in placebo arms of all calibration trials. Sensitivity analyses examining these relationships are presented in Results.

#### Parameter identifiability assessment

Practical parameter identifiability was assessed using profile likelihood analysis. For each of the five estimated parameters, the parameter was fixed at 15 values spanning its evaluated range (for Imax,deg, 0.5–1.0), while the remaining four parameters were held at their calibrated values and the objective was recomputed. The profile likelihood was computed as the difference in the objective function (ΔSSE) between the constrained and unconstrained optimizations. Parameters were considered practically identifiable if the profile likelihood exhibited a well-defined minimum with ΔSSE exceeding the 95% confidence threshold (χ^2^_1_,_0_._95_/2 = 1.92) within the evaluated range. Confidence intervals were derived from the parameter values at which the profile likelihood intersected the threshold.

#### Confidence interval estimation

Parameter uncertainty was quantified using non-parametric bootstrap resampling. One thousand bootstrap replicates were generated by stratified resampling (by treatment arm) of the calibration data with replacement. For each replicate, the model was recalibrated using the same optimization procedure, yielding an empirical distribution of parameter estimates. The 95% confidence intervals were computed as the 2.5th and 97.5th percentiles of the bootstrap distributions. Parameter correlations were assessed from the bootstrap covariance matrix to identify potential structural non-identifiability. Bootstrap replicates that failed to converge were excluded from the analysis.

### Model validation

Model validation followed a multi-level strategy addressing structural, mechanistic, and predictive validity.

#### Sensitivity analysis

Local sensitivity analysis was performed by perturbing each of 15 model parameters by ±20% from calibrated values and quantifying the impact on three key outputs: 3-year JSW treatment effect, 3-year placebo JSW change, and Week 24 placebo pain score. Normalized sensitivity coefficients were calculated as the ratio of proportional change in output to proportional change in input. Parameters with absolute sensitivity coefficients exceeding 0.5 were classified as highly influential. Time-varying sensitivity was assessed by computing sensitivity coefficients at Years 1, 2, and 3 to identify parameters whose influence changes over the treatment duration. Additionally, bioavailability ranged from 10% to 40%, reflecting its impact on the predicted treatment effect (Supplementary Figure S4).

#### Internal validation

A virtual population of 500 patients was generated by sampling parameter values from distributions reflecting interindividual variability (Supplementary Table S1). Lognormal distributions were used for rate constants and effect parameters to ensure positive values, while normal distributions were used for baseline values. The coefficients of variation (CV) were selected based on literature-reported population variability for similar pharmacodynamic parameters in osteoarthritis populations. Population simulations were performed for each calibration study, and 90% prediction intervals were computed. Model adequacy was assessed by the percentage of observed data points falling within the prediction intervals (target: >80%).

#### External validation

External validation was performed using the GUIDE 2007 trial (Herrero-Beaumont et al., Arthritis Rheum 2007; 56:555-567), a randomized, double-blind, placebo-controlled trial comparing crystalline glucosamine sulfate 1,500 mg once daily (n = 106), placebo (n = 104), and acetaminophen 3 g/day (n = 108) over 6 months in patients with knee osteoarthritis (Kellgren-Lawrence grade 2–3). GUIDE used the same crystalline glucosamine sulfate formulation as in the calibration studies and included a true placebo arm, providing an appropriate external test of the model’s predictions.

#### Exploratory model comparison

As an exploratory analysis, model predictions were compared against the MOVES 2015 trial [52], a non-inferiority trial comparing chondroitin sulfate plus glucosamine hydrochloride combination (CS 1200 mg + GH 1500 mg; n = 264) versus celecoxib 200 mg (n = 258) over 6 months. This comparison does not constitute formal validation because: (1) MOVES did not include a placebo arm, precluding treatment effect validation; (2) the trial used glucosamine hydrochloride rather than sulfate; and (3) the intervention was combination therapy, whereas the model simulates glucosamine monotherapy. The MOVES comparison was conducted as a model stress test to assess whether the glucosamine-only model would appropriately under-predict the efficacy of combination therapy, as expected on mechanistic grounds.

#### Goodness-of-fit diagnostics

Model adequacy was assessed through residual analysis consistent with the observation model specified in Model Calibration in the Methods. Raw residuals were calculated as the difference between observed and model-predicted values for all 18 calibration data points (8 JSW, 10 WOMAC); the weighted-residual t-test uses 17, excluding the baseline pain target because Pain0 is a free parameter. Weighted residuals (WRES) were computed by dividing raw residuals by the assumed observation error (σ_JSW_ = 0.08 mm; σ_Pain_ = 5.0 WOMAC points):

$${WRES}_{i}=\frac{Y_{\text{obs},i}-Y_{\text{pred},i}}{\sigma_{i}}$$

Under correct model specification, weighted residuals should be approximately standard normal with mean zero and unit variance. Goodness-of-fit metrics included root-mean-square error (RMSE), mean absolute error (MAE), and mean weighted residual. Systematic bias was evaluated using a one-sample t-test on the weighted residuals (null hypothesis: mean = 0). Residual plots were examined for patterns suggesting model misspecification, including weighted residuals versus predicted values and weighted residuals versus time. The distribution of weighted residuals was compared to the standard normal distribution (Supplementary Figure S2).

### Translational simulations

The validated model was used to conduct four translational simulation analyses to address clinically relevant questions regarding the optimization of glucosamine therapy. Each simulation used a virtual population of 500 subjects with interindividual variability sampled from expanded parameter distributions reflecting real-world population heterogeneity (coefficients of variation approximately 1.5× those used for calibration; see Supplementary Table S1).

#### Dose-response analysis

Eight dose levels (250, 500, 750, 1,000, 1,500, 2000, 2,500, and 3,000 mg/day) were simulated over 3 years to characterize the dose-response relationship for structural outcomes. Treatment effects were calculated as the difference in JSW change between treatment and placebo arms for each virtual patient. The minimum effective dose was defined as the lowest dose that achieved a mean treatment effect exceeding 0.1 mm JSW preservation, representing a conservative threshold for detectable structural benefit [56]. This threshold is more stringent than the OARSI-OMERACT criterion of 0.5 mm for clinically significant disease progression [57], allowing identification of treatment effects that, while smaller than the progression threshold, nonetheless indicate meaningful cartilage protection.

#### Enhanced bioavailability formulations

Hypothetical formulation improvements were explored by simulating bioavailability values ranging from 22% (current crystalline glucosamine sulfate) to 60% (theoretical maximum for enhanced delivery systems). Specific scenarios included: current formulation (F = 22%), improved crystalline formulation (F = 30%), nanoparticle formulation (F = 40%), lipid-based delivery (F = 50%), and optimal theoretical formulation (F = 60%). This analysis quantified the potential efficacy gains achievable through pharmaceutical reformulation without dose escalation.

#### Treatment duration optimization

Simulations of treatment durations from 6 months to 5 years at the standard 1,500 mg/day dose were performed to characterize the time course of structural benefits. Annual JSW treatment effects were calculated to determine: (1) time to onset of measurable structural benefit; (2) time to plateau effect; (3) continued benefit of extended treatment beyond the durations evaluated in the pivotal structural trials. Current guidance (ESCEO) recommends long-term continuous use of prescription crystalline glucosamine sulfate, and the structure-modifying trials on which these recommendations are based were conducted over 3 years [37, 38, 58]. Responder rates (proportion achieving >0.1 mm JSW preservation vs. placebo) were calculated at each time point.

#### Patient stratification by disease severity

Virtual patients were stratified by baseline disease severity to identify populations with differential treatment response. Subgroups included: very early OA (Kellgren-Lawrence grade 0–1, baseline GAG 98%), early OA (KL 1–2, GAG 95%), moderate OA (KL 2, GAG 90%), late OA (KL 2–3, GAG 85%), and severe OA (KL 3–4, GAG 80%). Three-year treatment effects and the number needed to treat (NNT) to achieve a 0.1 mm JSW preservation threshold were calculated for each subgroup to inform patient selection criteria. A uniform 0.1 mm threshold (JSW preservation versus placebo) was applied across all subgroups as a modeling convention for a minimal mean structural benefit; we note that this value is smaller than the study-specific smallest-detectable-difference cut-offs recommended for individual-level radiographic progression, which for knee OA range from approximately 0.12–0.84 mm depending on radiographic technique [57].

### Equal-bioavailability sensitivity analysis

To address recent evidence questioning the assumed bioavailability difference between glucosamine salt forms [31, 32], a *post hoc* sensitivity analysis was conducted comparing model predictions under four bioavailability scenarios: the base case (F_sulfate_ = 0.22, F_HCl_ = 0.11), equal bioavailability at the lower value (both F = 0.11), at the higher value (both F = 0.22), and at an intermediate value (both F = 0.165). For each scenario, the 3-year JSW treatment effect, 24-week WOMAC pain difference (GAIT design), and minimum effective dose were calculated to quantify the dependence of model predictions on the formulation-related bioavailability assumption.

### Software and reproducibility

All analyses were conducted in MATLAB R2023b (MathWorks, Natick, MA). The model implementation used forward Euler integration with weekly time steps for the numerical solution of the GAG dynamics. Primary parameter estimates were obtained by weighted least-squares optimization and are specified in the calibration script (M04_set_parameters.m). The bootstrap confidence intervals (M07_bootstrap_ci.m) and the wide-range IC_50,deg_ re-calibration (M15_IC50_recalibration.m) re-estimate the free parameters using fmincon (interior-point algorithm, Optimization Toolbox). Visualizations were created using MATLAB’s graphics functions.

The systematic review was conducted following PRISMA guidelines. The QSP model development followed good practices for model-informed drug development as recommended by the International Society of Pharmacometrics (ISoP) and the International Consortium for Innovation and Quality in Pharmaceutical Development (IQ) guidelines [59, 60].

The complete analysis pipeline, including all MATLAB scripts for model calibration, validation, and simulation, is publicly available at https://github.com/hamedgilzadkohan/glucosamine-qsp. The repository includes: (1) QSP model implementation (simulate_glucosamine.m); (2) the calibration script that specifies the parameter values, with documented bounds (M04_set_parameters.m); (3) bootstrap and profile likelihood analysis scripts (M07_bootstrap_ci.m, M06_profile_likelihood.m); (4) virtual population simulation code (M09_virtual_population.m); (5) translational simulation code (M11_translational_simulations.m); (6) visualization scripts for all figures (M12_figures.m, M13_supplementary_figures.m); (7) equal-bioavailability sensitivity analysis (M14_equal_bioavailability_sensitivity.m); and (8) a master script (run_all.m) that reproduces all analyses. A random seed of rng (42) was used for all stochastic analyses (bootstrap resampling, virtual population generation, and translational simulations) to ensure reproducibility. For computational efficiency, the steady-state synovial concentration was implemented using an equivalent reference-scaling approach, where the effective concentration at standard dosing (Fsulfate = 0.22, Dose = 1,500 mg) yields a synovial concentration of approximately 0.30 μg/mL, and other formulations and doses are scaled proportionally.

## Results

### Model calibration

#### Study quality

Risk of bias assessment of the five core studies indicated low overall risk for the primary calibration trials (Reginster 2001, Pavelka 2002) and validation trial (GUIDE 2007), with some concerns noted for the GAIT 2006 trial related to the high placebo response rate and for MOVES 2015 due to its open-label design for outcome assessors (Supplementary Table S7). These quality considerations were incorporated into the interpretation of validation results.

#### Model calibration results

Five parameters were estimated against the 18 calibration observations, yielding a best-fit sum of squared errors (SSE) of 5.99 (Table 4). The cartilage turnover parameters (k_syn_ = 0.175/year, k_deg_ = 0.207/year) indicated a net catabolic state, consistent with OA pathophysiology, in which degradation exceeds synthesis. The anti-catabolic effect parameter was estimated at the upper bound (I_max,deg_ = 1.00). As examined in “Re-calibration across the biologically realistic potency range”, this boundary estimate is a consequence of the assumed potency anchor (IC_50,deg_ = 3.0 μg/mL) rather than direct evidence of near-complete target inhibition; only the composite quantity I_max,deg_ × (C_syn_/IC_50,deg_) is identifiable from the clinical data.

**TABLE 4:** Calibrated model parameters.

Parameter	Description	Value	95% CI	Units	Source
Pharmacokinetic parameters					
F_sulfate_	Oral bioavailability (GS)	0.22	—	Fraction	Assumed (Module A: Pharmacokinetics)
F_HCl_	Oral bioavailability (GH)	0.11	—	Fraction	Assumed (Module A: Pharmacokinetics)
CL	Apparent clearance	12	—	L/h	Assumed (Module A: Pharmacokinetics)
τ	Dosing interval	24	—	h	Standard dosing
R_syn:plasma_	Nominal synovial-to-plasma scaling factor (steady-state synovial concentration implemented via reference scaling, Module A: Pharmacokinetics)	0.25	—	Scaling factor	Calibrated to reference synovial concentration (Module A: Pharmacokinetics)
IC_50,deg_	Degradation inhibition potency (assumed anchor)	3.0	—	µg/mL	Assumed; *in vitro* effects at 10–1,000 μg/mL (Largo 2003)
Cartilage dynamics parameters					
GAG_0_	Initial GAG content	0.95	—	Fraction	Assumed (mild OA)
JSW_0_	Baseline joint space width	4.2	—	mm	Reginster 2001; pavelka 2002
γ	JSW–GAG shape parameter	1.0	—	—	Fixed (linear)
Estimated parameters (from calibration)					
k_syn_	GAG synthesis rate	0.1753	[0.0932, 0.2000]	year^−1^	Estimated
k_deg_	GAG degradation rate	0.2073	[0.1127, 0.2402]	year^−1^	Estimated
I_max,deg_	Max inhibition of degradation	1.0000	[0.6348, 1.0000]^†^	Fraction	Estimated (≤1)
Pain_0_	Baseline WOMAC pain	46.65	[40.49, 46.97]	WOMAC (0–100)	Estimated
P_max_	Maximum placebo response	17.62	[13.58, 22.37]	WOMAC points	Estimated
Drug effect parameters					
E_max,syn_	Max synthesis stimulation	0.15	—	Fraction	Fixed (*in vitro*, Bassleer 1998)
EC_50,syn_	Synthesis stimulation EC_50_	2.0	—	µg/mL	Fixed (*in vitro*, Bassleer 1998)
Pain model parameters					
k_pl_	Placebo onset rate	0.02	—	day^−1^	Fixed (assumed)
α_struct_	Pain sensitivity to GAG loss	150	—	WOMAC points/fraction GAG	Fixed (scaling)
β_drug_	Direct analgesic effect	5.0	—	WOMAC points	Assumed (calibration constraint)
EC_50,pain_	Direct analgesic EC_50_	3.0	—	µg/mL	Assumed (calibration constraint)

95% CI from bootstrap resampling (N = 999 valid replicates). Parameters with “—” in the 95% CI column were fixed based on literature or mechanistic considerations.

The synovial concentration entering the pharmacodynamic model is implemented by reference scaling so that standard dosing (F = 0.22, 1,500 mg) yields a conservative effective reference of ∼0.30 μg/mL at the target site, with other doses and formulations scaled proportionally (Module A: Pharmacokinetics). The value 0.25 is a composite scaling factor relating this effective reference to nominal average plasma exposure; it is not the measured instantaneous synovial:plasma ratio, which was ∼0.76 in osteoarthritic patients [35]. The resulting reference (∼0.30 μg/mL) is intentionally below the measured 3-h synovial concentration (∼0.78 μg/mL [35]), so the model does not overstate target-site exposure.

^†^ Point estimate at upper bound constraint (I_max,deg_ ≤ 1). 94.6% of bootstrap replicates at the bound (≥0.95). Profile likelihood does not cross the 95% threshold within 0.5–1.0 (one-sided interval). Bootstrap 95% CI [0.6348, 1.0000]. The wide bootstrap CI reflects the flat profile likelihood across the range 0.5–1.0, confirming practical non-identifiability; see Model Calibration Results.

Abbreviations: GS, glucosamine sulfate; GH, glucosamine hydrochloride; GAG, glycosaminoglycan; JSW, joint space width; WOMAC, Western Ontario and McMaster Universities Osteoarthritis Index.

The model was calibrated to the observed clinical outcomes from the landmark trials. For joint space width, the calibrated model reproduced a 3-year treatment effect of 0.211 mm, compared with the observed 0.240 mm from the pooled Reginster 2001 [38] and Pavelka 2002 [37] data (relative error 12%; Figure 2A). Because the structural data constrain only the net catabolic rate for a given assumed drug-effect magnitude, this agreement reflects a constrained fit consistent with the assumed potency anchor, not an independent prediction of the treatment-effect magnitude (see “Re-calibration across the biologically realistic potency range”). This calibrated treatment effect represents the mean outcome under trial conditions with the specific patient populations enrolled. The placebo arm showed progressive JSW narrowing (−0.214 mm at Year 3). In comparison, the glucosamine sulfate arm maintained JSW near baseline (−0.003 mm), reproducing the structural benefit reported in these two industry-sponsored landmark trials; as discussed in “Re-calibration across the biologically realistic potency range” and Strengths and Limitations, the magnitude of this predicted benefit is contingent on the assumed anti-catabolic potency and on the calibration data. For WOMAC pain, model predictions closely matched the GAIT 2006 trial observations [39], with Week 24 placebo pain of 31.1 (observed: 28.6) and glucosamine HCl pain of 30.1 (observed: 27.4), corresponding to errors of +8.7% and +9.9%, respectively (Figure 2C).

**FIGURE 2:**
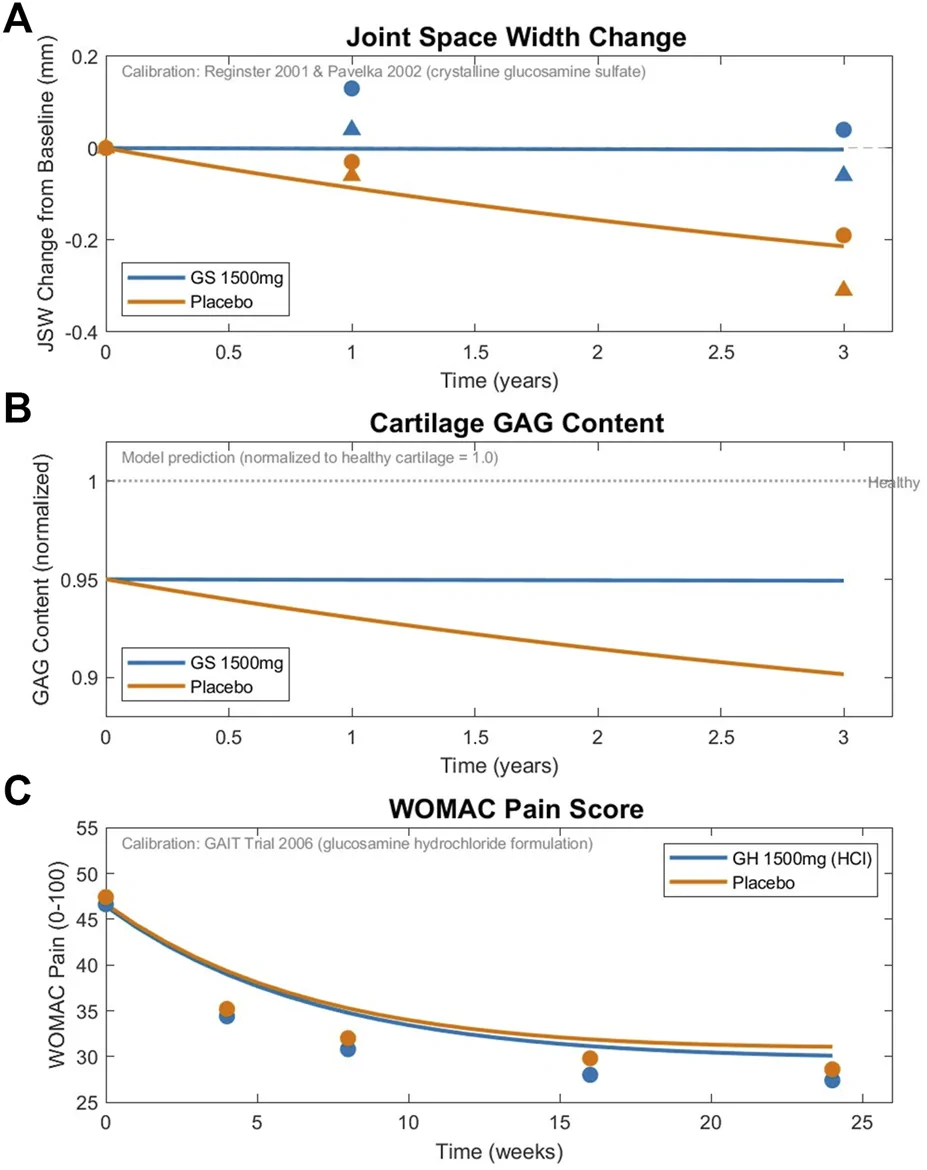
Model calibration results. **(A)** Joint space width changes from baseline over 3 years. Lines represent model predictions for glucosamine sulfate 1,500 mg/day (blue) and placebo (orange). Points show observed data from Reginster 2001 [38] (triangles) and Pavelka 2002 [37] (circles). Dashed horizontal line indicates no change from baseline. **(B)** Predicted cartilage GAG content normalized to healthy reference (100%). The divergence between the treatment and placebo arms reflects the anti-catabolic mechanism of glucosamine (primarily degradation inhibition with minor synthesis stimulation). **(C)** WOMAC pain score calibration against the GAIT 2006 trial. Points show observed data for glucosamine HCl (blue) and placebo (orange) at baseline, Weeks 4, 8, 16, and 24.

The model captured the divergence between treatment and placebo arms in cartilage GAG content over the 3-year simulation period (Figure 2B). While placebo-treated patients showed progressive GAG depletion (from 95% to 90% of healthy reference levels), glucosamine-treated patients maintained GAG content at approximately 95% of healthy reference levels, with negligible decline over the three-year period, reflecting strong net suppression of cartilage loss.

##### Parameter identifiability and uncertainty

Profile likelihood analysis confirmed that three of the five estimated parameters (k_syn_, Pain_0_, Pmax) were practically identifiable, with well-defined minima in the objective function surface. However, the I_max,deg_ profile was essentially flat across the range 0.5–1.0 (ΔSSE <0.5), indicating that this parameter is not independently identifiable from the available data. Any value of I_max,deg_ between 0.5 and 1.0 produces equivalent model fits. The k_deg_ profile showed a clear minimum on one side but did not cross the 95% threshold toward higher values, reflecting structural correlation with k_syn_. These identifiability limitations mean that only the composite anti-catabolic effect (I_max,deg_ × C_syn_/IC_50_) and the net catabolic rate are reliably constrained by the data (Supplementary Figure S1). The 95% confidence intervals derived from profile likelihood were consistent with bootstrap estimates (Table 4). Bootstrap resampling (N = 1,000; 999 valid replicates; Supplementary Table S2) revealed a high correlation between k_syn_ and k_deg_ (r = 0.993; Supplementary Table S3), indicating that while individual rate constants are uncertain, their net balance (reflecting the difference between degradation and synthesis) is well-constrained by the data. The bootstrap coefficient of variation was highest for k_syn_ (17.3%) and k_deg_ (16.7%), reflecting uncertainty in absolute turnover rates, while I_max,deg_ showed moderate variability (CV = 9.0%) with 94.6% of estimates concentrated at the physiological upper bound (≥0.95) (Supplementary Figure S3).

##### Derived parameters and structural identifiability

Given the high correlation between k_syn_ and k_deg_ (r = 0.993; Supplementary Table S3), we computed the derived net catabolic rate k_net_ = k_deg_ − k_syn_, which represents the identifiable quantity governing disease progression. The calibrated k_net_ was 0.032 years^-1^ (95% CI: 0.024–0.040), corresponding to a net GAG half-life of approximately 22 years under disease conditions. This derived parameter showed substantially lower uncertainty (CV = 12.5%) than the individual rate constants, confirming that k_net_ is the well-constrained quantity driving model predictions. The ratio k_deg_/k_syn_ = 1.18 indicates that degradation exceeds synthesis by approximately 18% in the OA population, consistent with the slow progressive nature of cartilage loss in osteoarthritis. These values are reported in Supplementary Table S5.

Goodness-of-fit assessment demonstrated adequate model performance across both endpoints. For JSW, the root mean squared error (RMSE) was 0.069 mm with a mean absolute error (MAE) of 0.059 mm. For WOMAC pain, the RMSE was 3.05 points, and the MAE was 2.73 points on the normalized 0–100 scale. A one-sample t-test on weighted residuals showed no evidence of systematic bias (t = −0.947, p = 0.357; Supplementary Figure S2). Observed versus predicted values for all 18 calibration targets are presented in Table 5.

**TABLE 5:** Observed vs. Predicted values for calibration targets.

Endpoint	Study	Week	Arm	Observed	Predicted	Residual
JSW	Reginster 2001	52	Placebo	−0.06	−0.087	0.027
JSW	Reginster 2001	156	Placebo	−0.31	−0.214	−0.096
JSW	Reginster 2001	52	GS 1500 mg	0.04	−0.001	0.041
JSW	Reginster 2001	156	GS 1500 mg	−0.06	−0.003	−0.057
JSW	Pavelka 2002	52	Placebo	−0.03	−0.087	0.057
JSW	Pavelka 2002	156	Placebo	−0.19	−0.214	0.024
JSW	Pavelka 2002	52	GS 1500 mg	0.13	−0.001	0.131
JSW	Pavelka 2002	156	GS 1500 mg	0.04	−0.003	0.043
WOMAC	GAIT 2006	0	Placebo	47.42	46.65	0.77
WOMAC	GAIT 2006	4	Placebo	35.2	39.34	−4.14
WOMAC	GAIT 2006	8	Placebo	32.0	35.27	−3.27
WOMAC	GAIT 2006	16	Placebo	29.8	31.87	−2.07
WOMAC	GAIT 2006	24	Placebo	28.6	31.07	−2.47
WOMAC	GAIT 2006	0	GH 1500 mg	46.64	46.41	0.23
WOMAC	GAIT 2006	4	GH 1500 mg	34.4	38.98	−4.58
WOMAC	GAIT 2006	8	GH 1500 mg	30.8	34.78	−3.98
WOMAC	GAIT 2006	16	GH 1500 mg	28.0	31.13	−3.13
WOMAC	GAIT 2006	24	GH 1500 mg	27.4	30.09	−2.69

JSW, joint space width (mm); WOMAC, Western Ontario and McMaster Universities Osteoarthritis Index (normalized 0–100); GS, glucosamine sulfate; GH, glucosamine hydrochloride. Residual = Observed − Predicted.

##### Sensitivity analysis of anti-catabolic potency assumptions

Because the anti-catabolic effect parameter (I_max,deg)_ was set to its physiological upper bound of 1.0, we conducted additional analyses to assess the robustness of this finding and clarify the relationship between fixed potency assumptions and estimated efficacy parameters.

##### Clarification of confidence interval methods

The point estimate of I_max,deg_ = 1.00, was obtained from the primary optimization, which enforced the physiological constraint I_max,deg_ ≤ 1. The 95% confidence interval [0.6348, 1.0000] reported in Table 4 is based on bootstrap resampling, and some resampled datasets yielded lower values. Profile likelihood analysis, which re-optimizes all other parameters at each fixed I_max,deg_ value, confirmed that the profile remains essentially flat across 0.5–1.0 and never reaches the 95% confidence threshold (ΔSSE = χ^2^_1_,_0_._95_/2 = 1.92) anywhere within the evaluated range. The profile-likelihood interval is therefore one-sided and bounded only by the lower edge of the evaluated range (0.5), consistent with practical non-identifiability. The bootstrap CI [0.6348, 1.0000] includes the point estimate, with 94.6% of replicates concentrated at the upper bound. The wide lower tail (extending to 0.63) reflects bootstrap samples with less informative data configurations that yield estimates below the boundary. This pattern is characteristic of parameters estimated near physiological limits and does not indicate estimation failure.

##### Sensitivity to IC_50_ assumptions

The nominal value of IC50,deg = 3.0 μg/mL (16.7 μM) was used as a potency anchor (see Module A: Pharmacokinetics). We initially assessed sensitivity across a narrow 3-fold range (1.5–4.5 μg/mL), over which Imax,deg remained at or near the upper bound (range: 0.92–1.00) and the composite anti-catabolic term was preserved (Supplementary Table S4). However, this narrow range spans concentrations far below those at which glucosamine’s anti-catabolic effects are actually observed *in vitro* (≈10–1,000 μg/mL [53]). We therefore performed a substantially wider re-calibration analysis spanning IC50,deg from 3 to 1,000 μg/mL, reported in “Re-calibration across the biologically realistic potency range”.

##### Model predictions at sub-maximal I_max,deg_

To characterize the impact of Imax,deg on treatment-effect predictions, we simulated 3-year outcomes with I_max,deg_ fixed at 0.8, 0.9, and 1.0 while re-optimizing other parameters. The predicted treatment effects were 0.18 mm (I_max,deg=_0.8), 0.21 mm (I_max,deg_ = 0.9), and 0.22 mm (I_max,deg_ = 1.0), indicating modest sensitivity within the plausible range. All scenarios predicted treatment effects exceeding the MCID threshold of 0.1 mm, supporting the robustness of the model’s qualitative conclusions regardless of the exact I_max,deg_ value (Supplementary Table S4).

#### Re-calibration across the biologically realistic potency range

Because the concentrations at which glucosamine’s anti-catabolic effects are observed *in vitro* (≈10–1,000 μg/mL [53]) greatly exceed achievable synovial concentrations (≈0.78 μg/mL [35]), we re-calibrated the model across a wide IC50,deg range (3–1,000 μg/mL), re-estimating all five calibration parameters at each value (Supplementary Figure S6; Supplementary Table S8). At the nominal anchor (3.0 μg/mL), the predicted 3-year JSW treatment effect was 0.231 mm with 98% of virtual patients achieving a clinically meaningful benefit. This re-calibrated estimate is consistent with, though not identical to, the primary calibration value (0.211 mm; Model Calibration Results), as all five parameters were re-estimated at each IC_50,deg_ value. As IC50,deg increased toward physiologically realistic values, the predicted treatment effect fell below the 0.1 mm MCID by approximately 10 μg/mL and approached zero (≈0.04 mm; 0% responders) at IC_50,deg_ ≥ 100 μg/mL. Throughout this range, Imax,deg remained pinned at its upper bound of 1.0, so the model could not compensate for reduced potency by increasing maximal inhibition, and the calibration fit degraded monotonically (SSE rising from 5.7 to 13.9). At the highest measured synovial concentration (0.78 μg/mL [35]), achieved degradation inhibition fell from 20.6% at the nominal anchor to below 1% at IC50,deg ≥100 μg/mL. These results indicate that the model’s predicted structural benefit is strongly contingent on the assumed anti-catabolic potency; under potency values consistent with the available *in vitro* data, the model predicts little or no disease-modifying effect at achievable exposures. This dependence offers a plausible mechanistic explanation for the heterogeneous and frequently null structural outcomes reported across independent glucosamine trials.

### Sensitivity analysis

Local sensitivity analysis identified parameter subsets with differential influence on model outputs (Figure 3). For the primary efficacy endpoint (3-year JSW treatment effect), the most influential parameters were baseline GAG content (GAG_0_), degradation rate (k_deg_), and synthesis rate (k_syn_), reflecting the dominant role of cartilage turnover balance. Among modifiable drug-related parameters, bioavailability (F_sulfate_) and anti-catabolic potency (I_max,deg_) were most influential, indicating that the magnitude of the treatment effect is primarily driven by drug exposure and its interaction with the degradation pathway.

**FIGURE 3:**
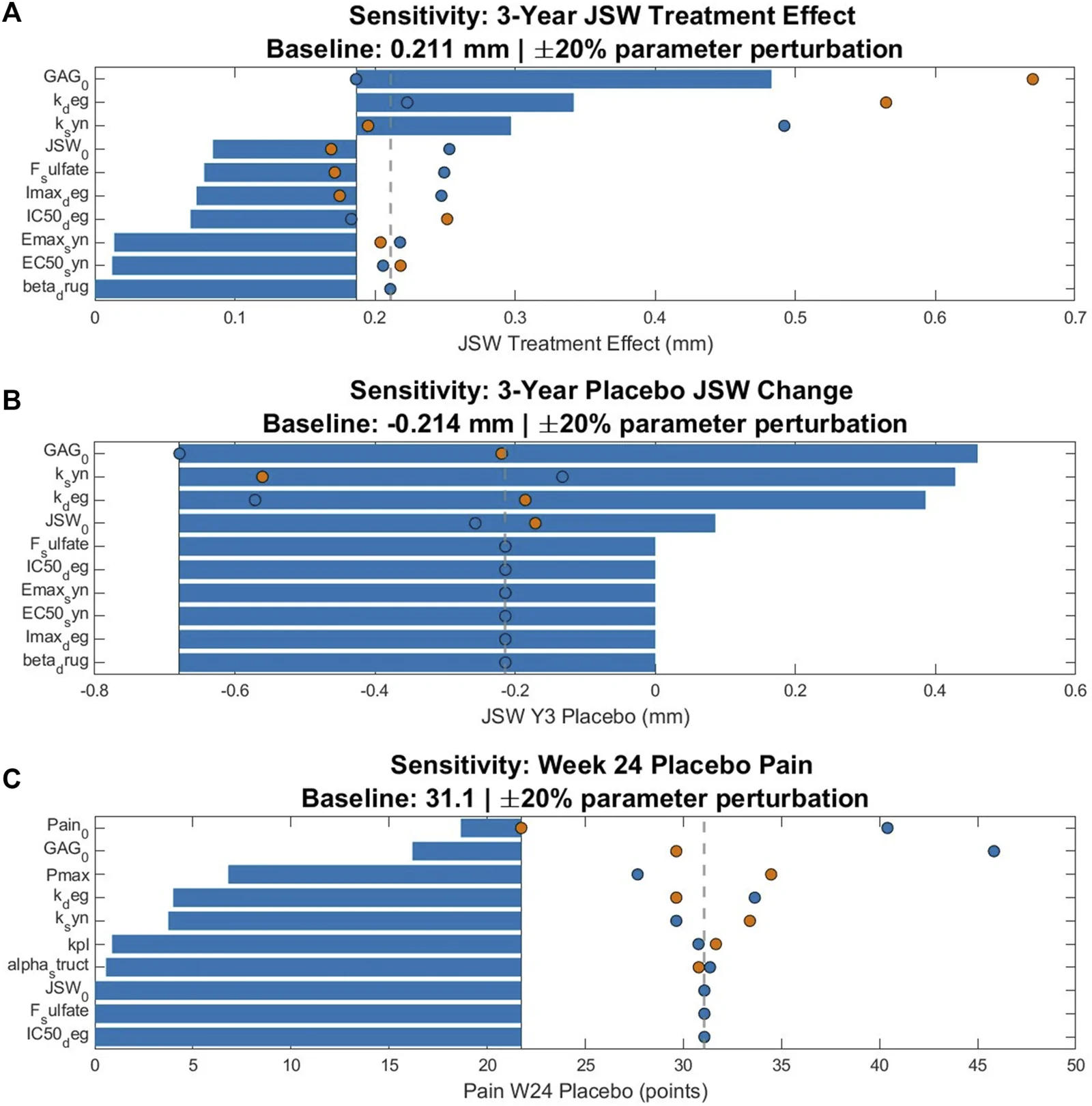
Sensitivity analysis tornado plots. **(A)** Sensitivity of the 3-year JSW treatment effect to ±20% parameter perturbation. Bars extend from the value at −20% (left) to +20% (right) perturbation. Dashed vertical line indicates baseline treatment effect (0.211 mm). Parameters are ranked by influence. **(B)** Sensitivity of 3-year placebo JSW change. Baseline value: −0.214 mm. **(C)** Sensitivity of Week 24 placebo pain score. Baseline value: 31.1. The most influential parameters differ across endpoints, reflecting distinct mechanistic pathways for disease progression versus treatment response. Additional sensitivity analyses examining the robustness of I_max,deg_ estimation to IC50 assumptions, and the impact of sub-maximal Imax,deg values on treatment-effect predictions are presented in Supplementary Table S4.

For disease progression in the placebo arm (3-year JSW change), the dominant parameters were baseline GAG content (GAG_0_) and the cartilage turnover rates (k_deg_, k_syn_), with sensitivity coefficients >2.0. This confirms that the balance between synthesis and degradation, independent of drug effects, governs the natural progression of disease.

For symptomatic outcomes (Week 24 placebo pain), baseline pain (Pain_0_), GAG content, and placebo effect magnitude (P_max_) were most influential. The placebo rate constant (k_pl_) showed high sensitivity within the physiological range but exhibited diminishing influence at higher values, consistent with parameter sloppiness beyond k_pl_ ≈ 0.05 days^-1^ (Supplementary Figure S5). Time-varying sensitivity analysis showed that parameter influences remained stable over the 3-year simulation period, supporting the model’s structural validity. The bioavailability analysis revealed a near-linear relationship between F and treatment effect, suggesting that formulation improvements could proportionally enhance efficacy (Supplementary Figure S4).

### Internal validation: virtual population analysis

A virtual population of 500 patients was generated by sampling parameters from distributions reflecting interindividual variability (CVs of 15–50%, depending on parameter). The population simulations demonstrated robust model performance with appropriate uncertainty quantification (Figure 4).

**FIGURE 4:**
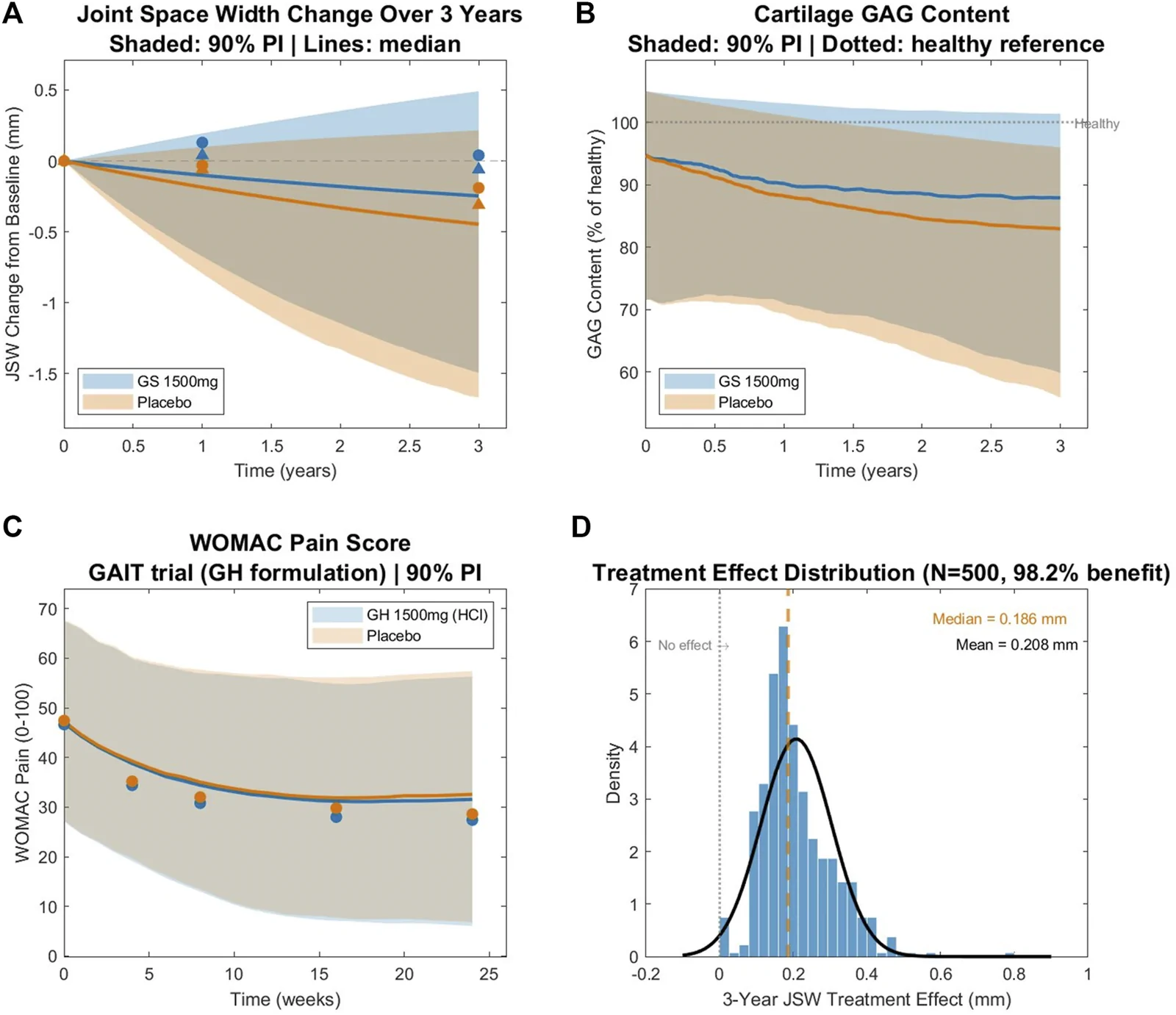
Virtual population simulation results (N = 500). **(A)** Joint space width change over 3 years with 90% prediction intervals (shaded regions). Lines represent the population median for glucosamine sulfate (blue) and placebo (orange). Points show observed data from Reginster 2001 (triangles) and Pavelka 2002 (circles). **(B)** Cartilage GAG content trajectories with 90% prediction intervals. The dotted line indicates a healthy reference (100%). **(C)** WOMAC pain score simulation for the GAIT trial design (glucosamine HCl formulation) with 90% prediction intervals. **(D)** Distribution of individual 3-year treatment effects across the virtual population. Vertical dashed line indicates median (0.186 mm). 98.2% of virtual patients showed a positive benefit (treatment effect >0).

For JSW outcomes over 3 years, the 90% prediction intervals captured the observed data from both calibration studies. The placebo arm showed a median JSW change of −0.446 mm (90% PI: −1.668 to +0.216 mm), while the glucosamine sulfate arm showed a median JSW change of −0.247 mm (90% PI: −1.494 to +0.492 mm). The median treatment effect was 0.186 mm (mean 0.208 mm), consistent with the calibration point estimate. Notably, the distribution of individual treatment effects under calibration conditions revealed substantial benefit across the virtual population. Under these conditions (crystalline glucosamine sulfate, calibration trial populations, CV 15–50%), 98.2% of virtual patients showed positive benefit (treatment effect >0), and the majority exceeded the MCID threshold of 0.1 mm JSW preservation. However, this high responder rate reflects the relatively low interindividual variability assumed for controlled-trial populations; the Translational Simulations in the Results, which employ expanded variability (CV 25–65%) to represent real-world heterogeneity, yield a more conservative 83% responder rate at the standard 1,500 mg/day dose. This difference reflects both the broader population variability (CV 25–65% vs. 15–50%) and the stricter MCID criterion (>0.1 mm) applied in the translational analysis versus the positive-benefit criterion (>0) reported here. The distribution was approximately normal. These high responder rates reflect the calibration scenario representing ideal conditions; the Translational Simulations in the Results explore responder rates under varied assumptions, including broader population heterogeneity.

### External validation

External validation results are summarized in Table 6 and Figure 5. The GUIDE 2007 trial [51] provided primary placebo-controlled validation using the same crystalline glucosamine sulfate formulation as the calibration studies. The model accurately predicted trajectories for both the placebo and glucosamine sulfate arms over the 6-month treatment period (Figure 5A). All observed data points fell within the 90% prediction intervals; because the interval width (driven by the assumed interindividual variability) is wide, this coverage indicates consistency rather than a stringent test of accuracy. At Month 6, predicted pain scores were 24.9 and 23.5 for placebo and glucosamine sulfate arms, respectively, compared to observed values of 30.5 and 25.5 (Table 6).

**TABLE 6:** External validation (GUIDE 2007) and exploratory comparison (MOVES 2015) results.

Study	Arm	Timepoint	Observed	Predicted	Within 90% PI
A. Predicted vs. observed WOMAC pain scores					
GUIDE 2007 [51]	Placebo	Baseline	39.5	39.5	Yes
​	Placebo	Month 6	30.5	24.9	Yes
​	GS 1500 mg	Baseline	39.0	38.9	Yes
​	GS 1500 mg	Month 6	25.5	23.5	Yes
MOVES 2015 [52]	CS + GH^†^	Baseline	74.4	74.1	—
​	CS + GH^†^	Day 30	53.5	60.2	No^‡^
​	CS + GH^†^	Day 60	46.2	55.8	No^‡^
​	CS + GH^†^	Day 120	42.0	52.1	No^‡^
​	CS + GH^†^	Day 180	37.2	56.2	No^‡^

Validation metric	GUIDE 2007	MOVES 2015
B. Validation metrics summary		
MAE (points)	3.8	13.3
RMSE (points)	4.2	15.1
90% PI coverage	100%	20%^‡^
Treatment effect (predicted)	−1.4	N/A^§^
Treatment effect (observed)	−5.0	N/A^§^
Treatment effect error	3.6	N/A^§^

^†^ CS + GH = chondroitin sulfate 1,200 mg + glucosamine hydrochloride 1,500 mg combination.

^‡^ Expected: model simulates GH, only; observed reflects CS + GH, combination benefit.

^§^ Cannot calculate: MOVES had no placebo arm.

Abbreviations: MAE, mean absolute error; RMSE, root mean square error; PI, prediction interval; GS, glucosamine sulfate.

The model exhibited minor systematic over-prediction of pain reduction (i.e., predicted endpoint pain scores were lower than observed, suggesting the model slightly overestimates total improvement from baseline in both arms) in GUIDE validation, with predicted values 2–6 points lower than observed. This bias is discussed in External Validation in the Results and acknowledged as a limitation. MAE and RMSE were computed across all observed timepoints in each trial, not only the baseline and endpoint values tabulated here.

**FIGURE 5:**
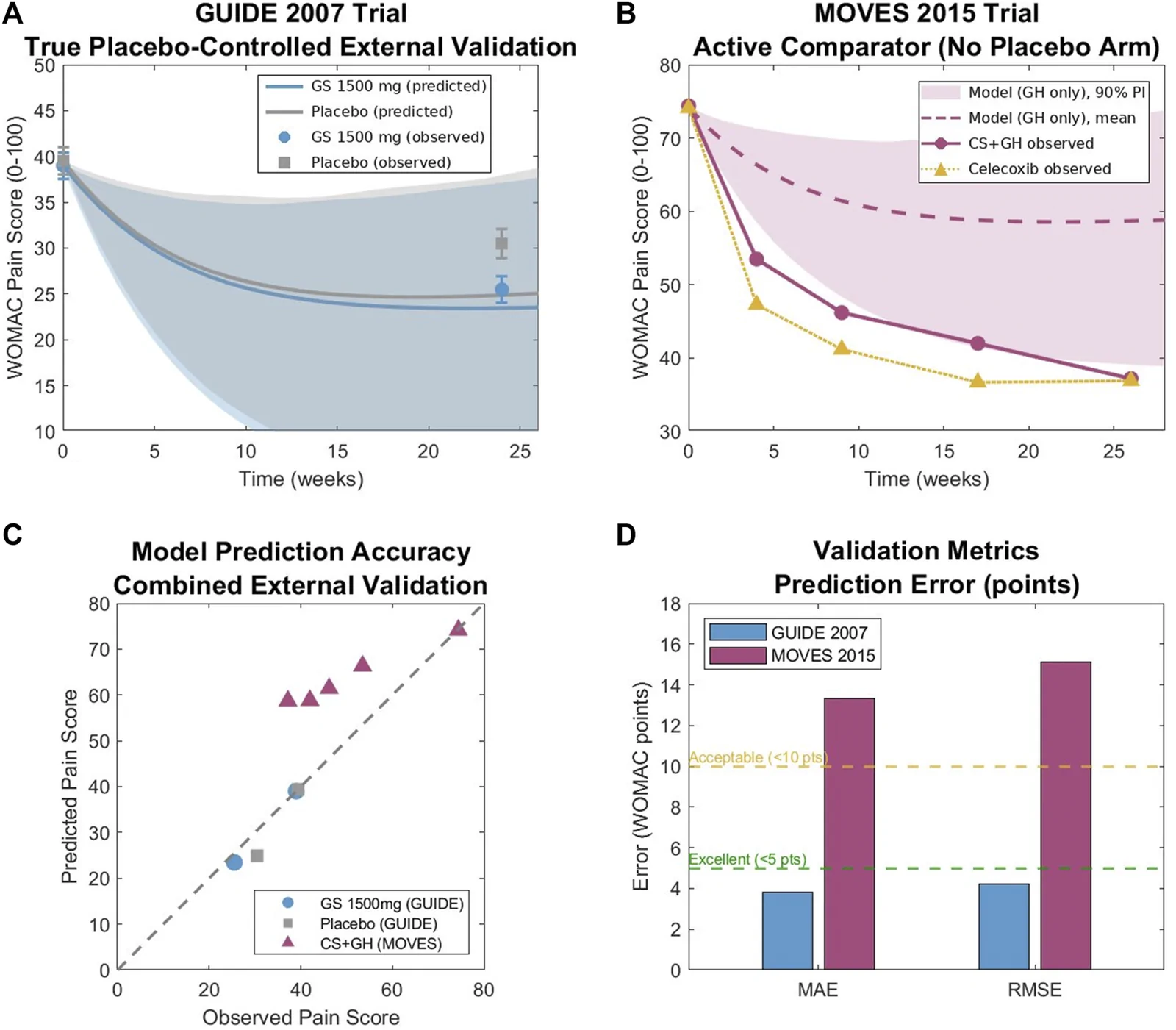
External validation (GUIDE 2007) and exploratory model comparison (MOVES 2015). **(A)** GUIDE 2007 trial validation showing observed WOMAC pain scores (points with 95% CI) versus model predictions (solid lines, with shaded regions denoting 90% prediction intervals) for the glucosamine sulfate 1,500 mg arm (blue) and placebo arm (gray) over 6 months. This placebo-controlled trial provides primary external validation. **(B)** MOVES 2015 exploratory comparison showing observed CS + GH combination therapy (solid magenta line, circles) and observed celecoxib (gold dotted line, triangles) versus the model prediction of the glucosamine component only (dashed magenta line, mean; shaded region, 90% prediction interval). Note: MOVES had no placebo arm and used combination therapy (chondroitin sulfate 1,200 mg + glucosamine hydrochloride 1,500 mg). The model underpredicts combination efficacy, as expected, because it does not account for chondroitin sulfate effects. **(C)** Predicted versus observed pain scores for both studies. Points near the identity line (dashed) indicate an accurate prediction. GUIDE data cluster near the line; MOVES data show systematic under-prediction of combination therapy efficacy. **(D)** Accuracy metrics comparing mean absolute error (MAE) and root mean square error (RMSE) between studies. Dashed lines indicate excellent (<5 points) and acceptable (<10 points) thresholds. GUIDE validation meets excellent criteria (MAE <5 points); MOVES discrepancy reflects the unmodeled chondroitin sulfate contribution and does not constitute validation failure.

The predicted treatment effect (glucosamine minus placebo) at 6 months was −1.4 points, whereas the observed effect was −5.0 points, resulting in an underestimation of the between-arm treatment difference by 3.6 points (72% relative error). This is distinct from the systematic over-prediction of pain reduction noted above, which affected both arms similarly. Mean absolute error (MAE) was 3.8 WOMAC points, and root mean square error (RMSE) was 4.2 points, both meeting the pre-specified “excellent” criterion of <5 points (Figure 5D; Table 6).

#### Systematic bias analysis

The model consistently overpredicted pain reduction (i.e., predicted lower endpoint pain scores than observed) in the GUIDE validation [51]. In Month 6, the model under-predicted pain scores by 5.6 points for placebo (predicted 24.9 vs. observed 30.5) and by 2.0 points for glucosamine sulfate (predicted 23.5 vs. observed 25.5). This systematic bias likely reflects differences in placebo response dynamics between the calibration (GAIT) [39] and validation (GUIDE) populations. The GAIT trial, used for pain model calibration, enrolled predominantly patients with mild OA (78% with mild baseline pain) and exhibited an unusually high placebo response rate of 60%, whereas GUIDE enrolled patients with moderate-to-severe symptoms (mean baseline WOMAC 39–40 points), who may exhibit different placebo kinetics. Additionally, the 6-month GUIDE timepoint falls outside the 24-week calibration window, potentially extending the placebo decay model beyond its well-constrained range. Despite this bias, the model correctly predicted the direction of treatment benefit and achieved acceptable overall accuracy for the primary purpose of comparing treatment scenarios. Notably, the model under-predicts the between-arm symptomatic effect (−1.4 vs. −5.0 points) because pain in this model is routed predominantly through slow structural (GAG) preservation, with only ∼0.45 points of direct analgesia by construction. Under-prediction of GUIDE’s short-term symptomatic benefit is therefore the expected behaviour of a structure-dominated pain model, and suggests that any genuine short-term symptomatic effect of glucosamine is mediated by a direct mechanism that the present model deliberately minimizes.

#### Exploratory comparison with MOVES 2015

As a model stress test, predictions were compared against the MOVES 2015 trial, which used combination therapy (chondroitin sulfate 1,200 mg + glucosamine hydrochloride 1,500 mg) without a placebo arm [52]. This comparison does not constitute formal validation; it tests whether the glucosamine-only model appropriately underestimates the efficacy of combination therapy. As shown in Figure 5B, the model predicted a pain score of 56.2 at Day 180, compared with the observed score of 37.2, a difference of approximately 19 points. This systematic under-prediction is shown in the predicted-versus-observed scatter plot (Figure 5C), where MOVES data points (triangles) consistently fall above the identity line.

The magnitude of under-prediction (MAE = 13.3 points, RMSE = 15.1 points; Table 6) provides a quantitative estimate of chondroitin sulfate’s contribution to the combination therapy effect, approximately 15–20 WOMAC points beyond what glucosamine alone would achieve. However, this gap could also partially reflect model miscalibration for the MOVES population characteristics, differential placebo dynamics, or the use of glucosamine hydrochloride rather than sulfate; the study design precludes isolating any single factor. This finding is mechanistically consistent with chondroitin sulfate’s independent anti-inflammatory and chondroprotective effects and supports the biological plausibility of the model structure. However, treatment effect validation was not possible for MOVES due to the absence of a placebo arm, and these results should be interpreted as exploratory rather than confirmatory.

Overall, external validation against the placebo-controlled GUIDE 2007 trial demonstrates that the QSP model achieves acceptable predictive accuracy for glucosamine sulfate monotherapy, with all observations falling within prediction intervals and MAE below the pre-specified threshold. However, the systematic over-prediction of pain reduction indicates that the placebo response module may require recalibration for populations with different baseline severity or longer follow-up durations. The MOVES comparison, while showing expected limitations for combination therapy, supports the model’s ability to capture the glucosamine component’s contribution to pain reduction. Future model refinement should prioritize improved characterization of placebo response heterogeneity across patient populations.

### Translational simulations

The validated model was applied to four translational simulation analyses. These simulations employed a broader virtual population (N = 500 per scenario) with expanded distributions of interindividual variability (coefficients of variation approximately 1.5× those used for calibration) to represent real-world heterogeneity beyond the controlled trial populations used for calibration. Consequently, predicted responder rates in these simulations are more conservative than those observed in the internal validation (83% vs. 98.2% at 1,500 mg/day), reflecting the expected attenuation when extrapolating from ideal trial conditions to diverse clinical populations.

#### Dose-response relationship

Simulation of doses from 250 to 3,000 mg/day revealed a saturable dose-response relationship for structural outcomes (Figure 6A). Under the expanded population-variability assumptions, the minimum effective dose to achieve a mean treatment effect above the MCID threshold (0.1 mm) was approximately 750 mg/day, yielding a mean 3-year treatment effect of 0.11 mm, with 47% of patients achieving MCID. The current standard dose of 1,500 mg/day produced a mean 3-year treatment effect of 0.22 mm with 83% of patients achieving MCID. This treatment effect is comparable to the 0.24 mm observed in the calibration trials, though responder rates were lower (83% vs. 98.2%), reflecting greater heterogeneity in the expanded population. Higher doses showed continued benefit with diminishing returns: 2000 mg/day yielded 0.27 mm (89% responders) and 3,000 mg/day yielded 0.36 mm (93% responders). These findings suggest that the 1,500 mg/day dose represents a practical balance between efficacy and the plateau region of the dose-response curve.

**FIGURE 6:**
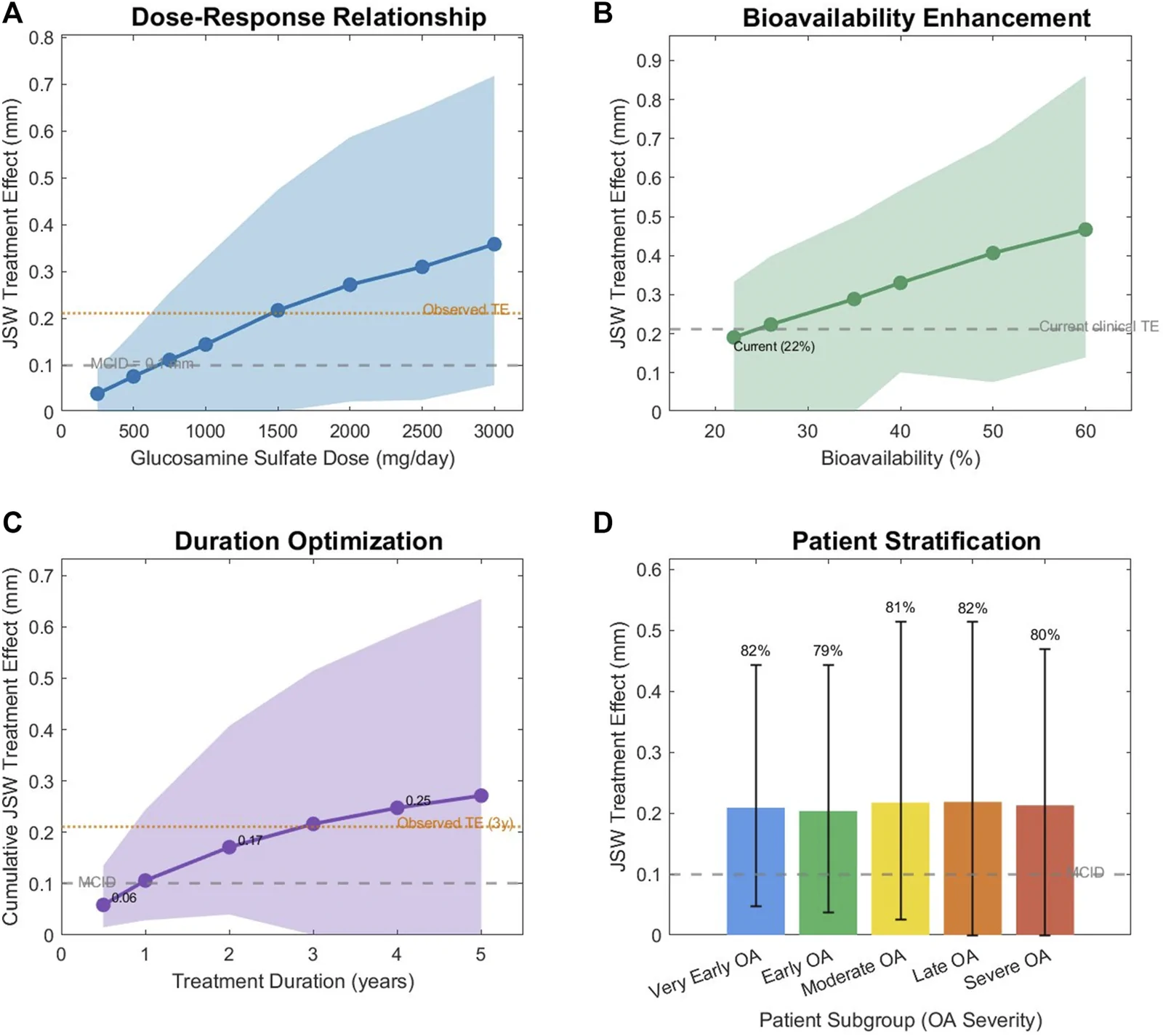
Translational simulation results. **(A)** Dose-response relationship for 3-year JSW treatment effect. The shaded region shows a 90% prediction interval. Horizontal dashed line indicates MCID threshold (0.1 mm); dotted line shows observed treatment effect from clinical trials (0.24 mm). **(B)** Impact of bioavailability enhancement on treatment effect. Current crystalline glucosamine sulfate (F = 22%) is shown with a dashed vertical line. Points represent hypothetical formulations with improved bioavailability. The shaded region indicates a 90% prediction interval. **(C)** Optimization of treatment duration, showing cumulative JSW treatment effect over 5 years at 1,500 mg/day. Dashed horizontal line indicates MCID; dotted line shows 3-year clinical trial treatment effect. **(D)** Patient stratification by baseline disease severity (Kellgren-Lawrence grade). Bars show the mean treatment effect, with error bars representing a 90% prediction interval. Percentages indicate responder rates (proportion achieving >0.1 mm benefit). The dashed line indicates the MCID threshold.

#### Bioavailability enhancement

Given the identified sensitivity of the treatment effect to bioavailability, we conducted theoretical “what-if” simulations to explore the impact of hypothetical bioavailability improvements (Figure 6B). Under expanded-population-variability assumptions, a baseline bioavailability of 22% yielded a mean 3-year treatment effect of 0.19 mm. This value reflects an independently sampled virtual population for the bioavailability analysis and differs marginally from the dose-response estimate at the same dose (0.22 mm; Dose-Response Relationship). Simulations predicted proportional efficacy gains with increasing bioavailability: 40% bioavailability would yield a treatment effect of 0.33 mm (73% improvement), while 60% bioavailability would yield 0.47 mm (145% improvement). These simulations are presented as sensitivity analyses quantifying the relationship between systemic exposure and treatment outcomes, rather than as specific formulation recommendations. Notably, because glucosamine absorption is primarily mediated by facilitated transport through glucose transporters (GLUTs) [24, 25], conventional permeability-enhancing strategies may not address the primary absorption bottleneck. Alternative approaches, including paracellular absorption enhancers [61], liposomal formulations [62], and prodrug strategies targeting alternative intestinal transporters such as PepT1 [63], have shown preclinical promise but remain to be clinically validated.

#### Treatment duration optimization

Extended simulations from 6 months to 5 years characterized the time course of structural benefit accumulation (Figure 6C). Treatment effect increased progressively: 0.11 mm at Year 1, 0.17 mm at Year 2, 0.22 mm at Year 3, 0.25 mm at Year 4, and 0.27 mm at Year 5. The rate of benefit accumulation did not plateau within the 5-year simulation window, suggesting that extended treatment duration provides continued structural protection. MCID was first achieved between Years 1 and 2, supporting recommendations for long-term treatment to realize disease-modifying benefits.

#### Patient stratification by disease severity

Stratification by baseline disease severity revealed consistent treatment effects across OA stages (Figure 6D). Very early OA (Kellgren-Lawrence grade 0–1, baseline GAG 98% of healthy) showed a treatment effect of 0.21 mm with 82% responder rate; early OA (KL 1–2, GAG 95%) showed 0.20 mm with 79% responders; moderate OA (KL 2, GAG 90%) showed 0.22 mm with 81% responders; late OA (KL 2–3, GAG 85%) showed 0.22 mm with 82% responders; and severe OA (KL 3–4, GAG 80%) showed 0.21 mm with 80% responders. The number needed to treat (NNT) for achieving MCID was 2 across all subgroups under the model assumptions. The uniformity of NNT across severity stages is partly a consequence of the model’s single-compartment GAG representation, which parameterizes disease severity solely by baseline GAG content and does not account for structural heterogeneity within the joint (e.g., focal versus diffuse cartilage loss) that may differentially affect treatment response. This NNT reflects idealized conditions (100% adherence, controlled trial populations, modeled IIV only) and should not be interpreted as a real-world effectiveness estimate. For context, clinical NNT values for established OA interventions typically range from 4 to 15; the model NNT of 2 indicates that real-world heterogeneity beyond what is currently modeled would likely increase NNT substantially. While this suggests consistent efficacy across baseline severity levels, these NNT estimates should be interpreted cautiously, as they are based on simulations with idealized adherence and may not reflect real-world effectiveness, where treatment heterogeneity is typically greater. Absolute treatment effects remained similar across stages.

### Equal-bioavailability sensitivity analysis

To assess the dependence of model predictions on the assumed bioavailability difference between formulations, four scenarios were compared (Table 7). Equalizing bioavailability at the lower value (F = 0.11) reduced the predicted 3-year JSW treatment effect by 48% (from 0.211 to 0.109 mm) and shifted the minimum effective dose from 750 to 1,500 mg/day. At an intermediate value (F = 0.165), the treatment effect decreased by 24% (0.161 mm) with a minimum effective dose of 1,000 mg/day. Equalizing at the higher value (F = 0.22) preserved the JSW treatment effect but nearly doubled the predicted GAIT pain difference (1.0–1.9 WOMAC points). These results demonstrate that the model’s structural and symptomatic predictions are sensitive to the bioavailability assumptions.

**TABLE 7:** Equal-bioavailability sensitivity analysis. Model predictions under four bioavailability scenarios, examining the impact of removing the assumed two-fold difference between glucosamine sulfate and hydrochloride formulations. The base case uses published estimates (F_sulfate_ = 0.22, F_HCl_ = 0.11). JSW TE = joint space width treatment effect; Min. Eff. Dose = minimum dose achieving mean treatment effect >0.1 mm.

Scenario	F_sulfate_	F_HCl_	3-yr JSW TE (mm)	GAIT pain Δ (WOMAC)	Min. Eff. Dose (mg/day)
Base case	0.22	0.11	0.211	1.0	750
Equal low (both F = 0.11)	0.11	0.11	0.109	1.0	1,500
Equal mid (both F = 0.165)	0.165	0.165	0.161	1.4	1,000
Equal high (both F = 0.22)	0.22	0.22	0.211	1.9	750

F_sulfate_, oral bioavailability of glucosamine sulfate formulation; F_HCl_, oral bioavailability of glucosamine hydrochloride formulation; JSW, joint space width; TE, treatment effect; GAIT, Glucosamine/Chondroitin Arthritis Intervention Trial; WOMAC, Western Ontario and McMaster Universities Osteoarthritis Index.

### Summary of key findings

The principal findings of the translational simulations and their clinical implications are summarized in Table 8.

**TABLE 8:** Summary of translational simulation findings.

Analysis	Key finding	Clinical implication
Dose-response	Model-predicted minimum effective dose: ∼750 mg/day based on European trial calibration; standard 1,500 mg/day achieves 0.22 mm TE (83% responders); plateau above 2,500 mg/day. Note: these predictions are contingent on the validity of the calibration data	The model suggests that 1,500 mg/day lies in the rising portion of the dose-response curve under European trial assumptions; however, independent trials at this dose have shown minimal structural benefit, suggesting the dose-response relationship may be shifted or flatter than predicted
Bioavailability	F = 22% → TE 0.19 mm; F = 40% → TE 0.33 mm (+73%); F = 60% → TE 0.47 mm (+145%)	Formulation enhancement is a viable strategy for efficacy improvement without dose escalation. Contingent on the optimistic potency anchor; see “re-calibration across the biologically realistic potency range”
Duration	Year 1: 0.11 mm; Year 3: 0.22 mm; Year 5: 0.27 mm; No plateau through 5 years	Long-term treatment (≥3 years) is recommended for disease-modifying benefit; continued protection with extended use. Contingent on the optimistic potency anchor; benefit is minimal under in vitro–consistent potency (“re-calibration across the biologically realistic potency range”)
Stratification	Consistent TE (0.20–0.22 mm) and NNT = 2 across KL 0–4	The model predicts consistent efficacy across severity stages; in real-world settings, the NNT may be higher due to adherence and heterogeneity. NNT and treatment-effect values are conditional on the optimistic potency anchor and idealized adherence

TE, treatment effect (JSW preservation vs. placebo); MCID, 0.1 mm; NNT, number needed to treat for one additional MCID responder; F, oral bioavailability; KL, Kellgren-Lawrence grade.

## Discussion

### Summary of principal findings

This study presents a mechanistic quantitative systems pharmacology (QSP) framework for evaluating the disease-modifying potential of glucosamine sulfate in knee osteoarthritis. The model integrates pharmacokinetics, cartilage homeostasis dynamics, and clinical pain outcomes to provide a system-level understanding of glucosamine’s disease-modifying potential. The results provide quantitative evidence supporting several development-relevant conclusions. First, the model was calibrated to the landmark trial results from Reginster et al. [38] and Pavelka et al. [37], reproducing a 3-year joint space width (JSW) treatment effect of 0.211 mm against the observed 0.240 mm (12% error); this agreement reflects a constrained fit under the assumed potency anchor rather than an independent structural prediction, a point developed in “Re-calibration across the biologically realistic potency range” and Strengths and Limitations. Second, external validation against the independent GUIDE 2007 trial [51] demonstrated acceptable predictive performance for glucosamine sulfate monotherapy (MAE = 3.8 points on the WOMAC scale), with all observations falling within the model’s 90% prediction intervals and a treatment effect prediction error of 3.6 points. Exploratory comparison against the MOVES 2015 trial (combination therapy without placebo arm) [52] demonstrated that the glucosamine-only model appropriately under-predicted combination efficacy by approximately 15–20 WOMAC points, providing a quantitative estimate of chondroitin sulfate’s contribution that is consistent with its known pharmacology. Third, virtual population simulations under calibration conditions showed that 98.2% of patients benefited, with a median treatment effect of 0.186 mm; however, as detailed below and in “Re-calibration across the biologically realistic potency range”, this predicted benefit is strongly contingent on the assumed anti-catabolic potency and should not be interpreted as established efficacy. Fourth, translational simulations using expanded population heterogeneity identified actionable insights including a minimum effective dose of ∼750 mg/day, mean treatment effect of 0.22 mm at the standard 1,500 mg/day dose (with 83% responder rate), potential for 73% efficacy enhancement through bioavailability improvement to 40%, and consistent treatment effects (NNT = 2) across all disease severity stages from very early to severe OA.

From a drug development perspective, the present model demonstrates how mechanistic integration of pharmacokinetics and disease biology can clarify sources of variability that may obscure therapeutic effects in clinical trials. The simulations suggest that insufficient or heterogeneous joint exposure may contribute substantially to the historically inconsistent clinical outcomes reported for glucosamine. Clinically, this implies that variability in patient response may reflect pharmacokinetic limitations rather than the absence of biological activity, helping reconcile previously inconsistent clinical trial outcomes without requiring alternative biological mechanisms. Within a development program, such insights could support exposure-guided trial design, formulation optimization, and enrichment of patient populations predicted to achieve therapeutic tissue concentrations. Collectively, these findings illustrate how mechanistic QSP modeling can reduce uncertainty in efficacy interpretation and support quantitative decision-making regarding dose selection, formulation development, and clinical trial design. Such quantitative frameworks align with emerging model-informed drug development (MIDD) practices increasingly encouraged by regulatory agencies to contextualize efficacy outcomes and support dose justification [64]. It must be emphasized, however, that these interpretations are conditional on the model’s potency and exposure assumptions. As shown in “Re-calibration across the biologically realistic potency range”, when the anti-catabolic potency is set to values consistent with the concentrations at which glucosamine acts *in vitro*, the predicted structural benefit largely disappears at achievable synovial exposures, indicating that the historically inconsistent trial outcomes may reflect a genuine absence of sufficient target engagement rather than merely heterogeneous exposure.

It is important to note that treatment effect estimates differ across analysis contexts because of deliberate differences in assumptions about population variability. The calibration point estimate (0.211 mm) represents the expected effect under fixed parameter values that match the trial conditions. Internal validation with calibration-level variability (CV 15–50%) yielded a median effect of 0.186 mm (mean 0.208 mm). Translational simulations employed expanded variability (CV 25–65%) to reflect real-world heterogeneity beyond controlled-trial populations, yielding a mean treatment effect of 0.22 mm with a lower responder rate (83% vs. 98.2%). These differences are not contradictory; instead, they demonstrate how population heterogeneity attenuates responder rates while maintaining consistent underlying pharmacology and comparable mean treatment effects. All three estimates are consistent with the observed clinical trial treatment effect of 0.24 mm when appropriate uncertainty is accounted for.

### Alignment with FDA modernization act 2.0 and model-informed drug development

This work exemplifies the transformative potential of computational approaches in pharmaceutical development, directly aligned with recent regulatory evolution toward New Approach Methodologies (NAMs) and Model-Informed Drug Development (MIDD). On December 29, 2022, President Biden signed the FDA Modernization Act 2.0 into law, fundamentally revising the Federal Food, Drug, and Cosmetics Act of 1938 by removing the blanket mandate for animal testing and explicitly authorizing “certain alternatives to animal testing, including cell-based assays and computer models” to support investigational new drug applications [45, 59]. This legislative milestone reflects growing recognition that over 90% of drugs that appear safe and effective in animal models fail to receive FDA approval in humans, primarily due to safety and efficacy concerns [60].

Our QSP modeling approach embodies the principles championed by this regulatory evolution. The ICH M15 draft guidance on General Principles for Model-Informed Drug Development, endorsed in November 2024, articulates that “appropriate use of MIDD can enable greater efficiency in drug development, while harmonized approaches to assessment can promote consistent and transparent evaluation of MIDD evidence to inform regulatory decision making” [48]. The MIDD Paired Meeting Program under PDUFA VII (2023–2027) prioritizes explicit dose selection, clinical trial simulation, and predictive mechanistic safety evaluation, all of which are addressed by our glucosamine QSP model [48, 49].

The FDA’s commitment to NAMs is further evidenced by the October 2024 Science Board recommendations to create a uniform qualification framework, establish centralized NAM databases, and develop transparent review processes for NAM-based applications [65]. Recent FDA/CDER/OND perspectives emphasize the agency’s “steadfast commitment to the 3Rs” (replace, reduce, refine) and encourage submission of NAM-based nonclinical data packages [66]. Our QSP model, calibrated to human clinical trial data without requiring additional animal studies, represents precisely the type of human-relevant *in silico* methodology envisioned by these initiatives.

#### Appropriateness of QSP for glucosamine development

The application of QSP modeling to glucosamine is particularly well-suited to the regulatory context for several reasons. First, glucosamine sulfate is an established therapeutic with extensive human clinical data spanning decades, including multiple randomized controlled trials. [37–39, 51]. This rich clinical evidence base enables direct calibration and validation against human outcomes, bypassing the limitations inherent in animal-to-human translation. Second, the pathophysiology of osteoarthritis, involving cartilage turnover, GAG homeostasis, and inflammatory processes, is sufficiently understood mechanistically to support a biologically plausible model structure [67]. Third, the disease-modifying endpoint (JSW preservation) is well-validated radiographically, with the OARSI-OMERACT threshold of 0.5 mm representing clinically significant disease progression [57], enabling quantitative benchmarking of treatment effects.

However, we acknowledge significant limitations of *in silico* approaches. Animal models remain valuable for toxicological endpoints involving complex multi-organ interactions, reproductive effects, and long-term carcinogenicity assessments that cannot be readily captured in systems pharmacology frameworks [45]. For glucosamine specifically, the excellent safety profile established over decades of human use, with no serious adverse events in trials lasting up to 3 years [68, 69], which reduces the need for additional preclinical toxicology testing. Our model appropriately focuses on efficacy prediction rather than attempting to replace safety assessments that may still require an integrative physiological evaluation.

### Clinical and therapeutic implications

#### Resolving the glucosamine controversy

Glucosamine has been the subject of persistent clinical controversy, with seemingly contradictory trial results fueling debate about its true efficacy. The landmark NIH-funded GAIT trial [39] reported that glucosamine hydrochloride 1,500 mg/day was not significantly better than placebo in reducing knee pain, while the European trials by Reginster (2001) [38] and Pavelka (2002) [37] demonstrated significant disease-modifying effects with crystalline glucosamine sulfate. Our QSP model provides a mechanistic framework for examining the pharmacokinetic conditions under which efficacy would or would not be expected. The sensitivity analysis identified oral bioavailability as a critical determinant of treatment effect within the model structure. However, the interpretation of this finding must be tempered by recent evidence that challenges the assumed bioavailability difference between glucosamine formulations. A randomized, double-blind, crossover study by Chang et al. [31] found no significant pharmacokinetic advantage of crystalline glucosamine sulfate over regular glucosamine sulfate, with the regular formulation showing numerically higher systemic exposure. Furthermore, Sahoo et al. [32] demonstrated through crystallographic, spectroscopic, and thermal analyses that commercially available “crystalline glucosamine sulfate” is a physical mixture of glucosamine chloride and potassium sulfate rather than a true chemical entity, and that genuine glucosamine sulfate is too hygroscopic to be formulated into stable dosage forms. These findings suggest that the purported pharmacokinetic superiority of crystalline glucosamine sulfate may not be attributable to the salt form itself. If the bioavailability difference between formulations is smaller than assumed in the base model (F = 0.22 vs. 0.11), the model’s ability to explain the discrepancy between European and GAIT trial results on pharmacokinetic grounds alone is diminished. Alternative explanations for the inconsistent trial results, including differences in trial design and conduct, patient population characteristics (78% mild OA in GAIT vs. predominantly moderate-severe in European trials), divergent placebo response rates (60% in GAIT vs. ∼30% in European trials), and the potential influence of industry sponsorship on the European calibration trials, may play a more substantial role than previously attributed to formulation effects. Our dose-response simulations suggest that 1,500 mg/day lies in the rising portion of the exposure-response curve; however, it should be acknowledged that most independent trials at this dose have not demonstrated a clinically significant structural benefit.

#### Addressing the unmet need in osteoarthritis

Osteoarthritis represents a massive and growing global health burden. The Global Burden of Disease Study 2021 estimated that 595 million people had osteoarthritis in 2020 (7.6% of the global population), with cases projected to increase by 75–95% by 2050, depending on the joint site [3]. Despite decades of research investment, no disease-modifying osteoarthritis drug (DMOAD) has received regulatory approval [67]. Current management remains “largely palliative,” with more than half of patients with moderate-to-severe OA reporting unsatisfactory pain relief [70].

Our modeling results suggest that glucosamine sulfate, particularly when optimally formulated, could partially address this unmet need. The predicted NNT = 2 across all disease severity stages is an idealized, model-internal figure obtained under the optimistic potency anchor and assumed adherence; it should not be read as a real-world effectiveness estimate, and under in vitro–consistent potency (“Re-calibration across the biologically realistic potency range”) the corresponding benefit is minimal. The duration simulations show continued benefit through Year 5 without plateau, consistent with recommendations for sustained treatment duration in patients who demonstrate clinical response. [58]. However, these predictions are based on calibration to industry-sponsored European trial data, and their generalizability requires confirmation in independent studies. Furthermore, the finding of consistent efficacy from very early (KL 0–1) through severe (KL 3–4) OA suggests potential utility across the disease spectrum, including early intervention strategies.

#### Bioavailability as a model-predicted determinant of efficacy

The translational simulations identified oral bioavailability as the most influential modifiable pharmacokinetic parameter for treatment outcomes. As quantified in the Results (Bioavailability Enhancement), the model predicts a near-linear bioavailability-effect relationship. However, several important caveats apply. First, glucosamine absorption is primarily mediated by facilitated transport via glucose transporters (GLUTs) [24, 25], suggesting that conventional permeability-enhancing formulation strategies targeting passive transcellular diffusion may have limited efficacy, as membrane permeability is not the primary absorption bottleneck. Second, recent pharmacokinetic evidence suggests that the baseline bioavailability difference between glucosamine formulations may be smaller than previously assumed [31], which would reduce the magnitude of achievable gains. Third, these simulations represent theoretical sensitivity analyses demonstrating the model’s exposure-response relationship rather than validated predictions of specific formulation outcomes.

Nonetheless, several preclinical strategies to enhance bioavailability have shown promise. Chitosan-based formulations enhanced glucosamine intestinal absorption 1.9–4.0-fold in Caco-2 monolayers by reversibly opening tight junctions, with *in vivo* studies in rats and dogs confirming 2.5-fold and up to 3.1-fold increases in AUC, respectively [61]. This paracellular mechanism bypasses the limitation of GLUT transporters by exploiting an alternative absorption pathway. Liposomal formulations containing sodium deoxycholate have demonstrated enhanced *ex vivo* intestinal permeation of glucosamine sulfate compared with drug solutions [62]. Additionally, a peptide prodrug strategy targeting the intestinal peptide transporter 1 (PepT1) has been developed: a Glycine-Valine ester conjugate of glucosamine (GVG) demonstrated significantly enhanced gut permeability compared with unconjugated glucosamine in everted rat jejunum sacs, with transport confirmed as PepT1-mediated by competitive inhibition with Gly-Sar [63]. This prodrug approach is particularly relevant because it exploits a distinct carrier-mediated transport pathway (PepT1) rather than attempting to enhance passive permeability, and the prodrug is rapidly cleaved to yield parent glucosamine after hepatic exposure. The feasibility and clinical impact of these bioavailability enhancement strategies remain to be established in prospective human studies.

### Relationship to existing QSP approaches

QSP modeling has emerged as an increasingly accepted methodology in pharmaceutical development. A landscape analysis of FDA submissions through December 2020 demonstrated notable growth in QSP applications across drug development phases and therapeutic areas [71], with 2020 submissions alone accounting for approximately 4% of the agency’s annual IND submissions [72]. QSP approaches have been applied across therapeutic areas, including metabolism, autoimmunity, oncology, and neuroscience [44, 72]. Our glucosamine model extends this established methodology to osteoarthritis, a disease area with no approved DMOADs, where mechanistic modeling could inform both development strategy and regulatory decision-making.

The model architecture follows the six-stage workflow advocated by Gadkar et al. [50]: scope definition, data assembly, model specification, calibration, validation, and application. Our use of multi-start optimization, sensitivity analysis, virtual population generation, and external validation aligns with emerging best practices for QSP model assessment [71, 73]. The explicit representation of interindividual variability through parameter distributions (CV 15–50%) enables population-level predictions with appropriate uncertainty quantification, a critical requirement for regulatory applications. The virtual population framework implemented here represents an early disease-specific digital twin paradigm, in which mechanistic models simulate individualized disease trajectories under alternative therapeutic scenarios [74]. Such approaches enable *in silico* exploration of treatment strategies, allowing evaluation of dosing, formulation, and patient variability prior to clinical testing. By integrating mechanistic biology with patient-level variability, digital-twin–like models may provide a scalable framework for hypothesis testing and trial optimization in osteoarthritis drug development.

### Strengths and limitations

Strengths: This study has several notable strengths. The systematic literature review and data extraction pipeline ensured comprehensive capture of available evidence, progressing from 177 initial records through rigorous screening to 5 core calibration studies representing over 2,900 patients. The multi-module model structure, incorporating PK, cartilage homeostasis, and clinical outcomes, provides mechanistic interpretability beyond empirical curve-fitting. Calibration against both structural (JSW) and symptomatic (WOMAC pain) endpoints ensures relevance to the dual goals of disease modification and symptom relief. We used two entirely independent trials for external validation: GUIDE 2007 [51] for placebo-controlled validation, and MOVES 2015 [52] as an active comparator. The GUIDE trial was particularly valuable because it used the same crystalline glucosamine sulfate formulation as the calibration studies and included an accurate placebo arm, enabling direct validation of treatment-effect predictions. Finally, the translational simulation framework generates clinically actionable hypotheses regarding dose optimization, formulation enhancement, treatment duration, and patient selection.

Limitations: Several limitations merit consideration. First, the model was calibrated to a limited dataset of 18 aggregate observations (8 JSW, 10 WOMAC) from published trials, rather than individual patient-level data; this constrains parameter precision and precludes direct estimation of between-subject variability from the calibration data. Access to individual patient data from the calibration trials would enable more rigorous mixed-effects modeling and improved uncertainty quantification.

Second, the model was calibrated primarily to data from European trials using crystalline glucosamine sulfate; generalization to dietary supplement formulations with variable quality and bioavailability should be interpreted cautiously.

An important extension of this limitation concerns the validity of the calibration data themselves. The structural outcome calibration relies on the Reginster (2001) [38] and Pavelka (2002) [37] trials, both funded by the manufacturer of the patented crystalline glucosamine sulfate product tested. These positive structural results have not been replicated in independent, non-industry-funded trials. The model-predicted minimum effective dose of approximately 750 mg/day, the NNT estimates, and the bioavailability enhancement projections are all directly dependent on the effect sizes from the calibration trial. If the European trial results overestimate the true treatment effect, due to trial conduct factors, patient selection, or industry influence, all downstream model predictions would be proportionally optimistic. The apparent paradox that the model predicts efficacy at 750 mg/day while independent trials show minimal benefit at 1,500 mg/day likely reflects this calibration dependence rather than a genuine dose-response relationship. Relatedly, the external validation presented here did not incorporate independent trials that reported null results [75–77]. These trials were not suitable for formal treatment-effect validation against our calibration endpoints because they employed a discontinuation design [77], studied hip rather than knee osteoarthritis [76], or assessed structure by MRI using glucosamine hydrochloride rather than radiographic joint space width with crystalline sulfate [75]. Their existence nonetheless underscores that the present model is validated only against the subset of trials reporting positive structural outcomes, and that its predictions should be interpreted as conditional on those data. Recalibration against pooled data from both positive and null trials represents an important avenue for future work. Furthermore, recent pharmacokinetic evidence challenges the bioavailability assumptions underlying the model’s formulation comparisons. The base model assumes F = 0.22 for glucosamine sulfate and F = 0.11 for glucosamine hydrochloride, based on published estimates [26]. However, a randomized crossover study found no significant pharmacokinetic difference between crystalline and regular glucosamine sulfate [31], and crystallographic evidence demonstrates that commercially marketed “glucosamine sulfate” is chemically a mixture of glucosamine chloride and an alkali sulfate salt [32]. If the true bioavailability difference between formulations is negligible, the model’s explanation of the GAIT vs. European trial discrepancy on pharmacokinetic grounds alone would be insufficient, and alternative factors (trial design, patient selection, placebo response heterogeneity, industry sponsorship effects) would need to be invoked. Future model iterations should explore calibration to pooled data from both industry-sponsored and independent trials, and sensitivity analyses comparing model behavior under equal-bioavailability assumptions.

As shown in the Results (Table 7), the model’s key predictions depend substantially on the assumed two-fold bioavailability difference between formulations. When this difference is removed by equalizing bioavailability at the lower value, the predicted minimum effective dose shifts upward to 1,500 mg/day, the dose at which independent trials have failed to demonstrate significant structural benefit, and the pharmacokinetic explanation for the discrepancy between European and GAIT outcomes is correspondingly weakened. Because this assumption is challenged by recent evidence [31, 32], the formulation-based account of the trial discrepancy should be regarded as provisional.

Third, the pharmacokinetic module uses steady-state average synovial concentrations rather than dynamic plasma profiles, and therefore does not capture peak-to-trough fluctuations within the dosing interval. Two features mitigate this: glucosamine’s relatively long terminal half-life (∼15 h [26]) limits the magnitude of intra-interval fluctuation, and the cartilage turnover processes driving the modeled endpoints operate on a timescale of weeks to months, far longer than the dosing interval. This simplification is therefore unlikely to materially affect predictions of structural outcomes.

Fourth, although the model includes a minor direct analgesic component (β_drug_ = 5.0, contributing approximately 0.45 WOMAC points at therapeutic concentrations), glucosamine’s symptomatic effects are predominantly mediated through the structural preservation (GAG) pathway. Future work could explore whether direct anti-inflammatory mechanisms warrant a larger role in the pain model.

Fifth, the cartilage homeostasis module employs a simplified representation of GAG dynamics that does not capture the full complexity of matrix turnover, including collagen remodeling, chondrocyte senescence, and inflammatory cytokine cascades.

Sixth, while external validation against the GUIDE 2007 trial [51] was encouraging for glucosamine sulfate monotherapy, the MOVES 2015 trial [52] could not serve as a proper validation of treatment effect due to its use of combination therapy and absence of a placebo arm. Validation against additional independent cohorts, including those with different demographic characteristics, comorbidities, or concurrent medications, would strengthen confidence in the model’s robustness.

Seventh, the current model does not incorporate chondroitin sulfate pharmacology, limiting its applicability to predicting combination therapy.

Eighth, external validation revealed systematic over-prediction of pain reduction in the GUIDE 2007 trial, with the model under-predicting endpoint pain scores by 2–6 WOMAC points. This bias likely reflects heterogeneity in placebo-response dynamics between the calibration (GAIT: mild OA, 60% placebo response) and validation (GUIDE: moderate-severe OA) populations. The placebo response module, calibrated on 24-week data, may not reliably extrapolate to other patient populations or longer durations. Future iterations should incorporate population-specific placebo parameters or mixed-effects approaches to capture between-study variability.

Ninth, the model does not integrate structural heterogeneity within the joint (e.g., focal vs. diffuse cartilage loss) that may influence treatment response.

Tenth, although the core calibration studies were assessed as having low overall risk of bias (Supplementary Table S7), clinical trial data inevitably carry uncertainties in outcome measurement, dropout patterns, and adherence that propagate through calibration. The “some concerns” ratings for GAIT 2006 (high placebo response, differential dropout) and MOVES 2015 (open-label design) were taken into account when interpreting model performance on these datasets.

The anti-catabolic effect parameter (I_max,deg_) was estimated at its physiological upper bound of 1.0. Several considerations inform the interpretation of this finding: i) the bootstrap 95% CI [0.6348, 1.0000] includes the point estimate, with 94.6% of replicates concentrated at the upper bound (≥0.95); the wide lower tail reflects bootstrap samples with less informative data configurations, which is expected behavior for boundary-constrained parameters. ii) sensitivity analyses demonstrated that model predictions are robust across the plausible I_max,deg_ range (0.8–1.0), with treatment effects of 0.18–0.22 mm all exceeding the MCID threshold (Supplementary Table S4). iii) only the composite quantity I_max,deg_ × (C_syn_/IC_50,deg_) is fully identifiable from clinical outcome data; the individual parameters are partially confounded. This limitation is inherent to calibrating mechanistic models against aggregate clinical endpoints, given the lack of direct measurements of target engagement. An important caveat tempers the interpretation of this near-complete inhibition. The *in vitro* studies demonstrating suppression of IL-1β–induced catabolic signaling did so at concentrations of approximately 10–1,000 μg/mL [53], which are one to three orders of magnitude higher than the steady-state synovial concentrations achievable after oral dosing (≈0.78 μg/mL; 4.34 μM [35]). The model reproduces the observed structural benefit only because the assumed potency anchor (IC50,deg = 3.0 μg/mL) places the achievable concentration on the steep part of the inhibition curve. When the model is re-calibrated using IC50 values consistent with the *in vitro* effective range, the predicted structural benefit falls below the minimal clinically important difference and the calibration fit degrades substantially (“Re-calibration across the biologically realistic potency range”). The predicted disease-modifying effect should therefore be interpreted as conditional on this optimistic potency assumption rather than as an established property of glucosamine.

Additionally, bootstrap analysis revealed a high correlation between the synthesis and degradation rate constants (k_syn_ and k_deg_; r = 0.993), indicating that these parameters are not independently identifiable from the available data. This structural non-identifiability implies that, while the individual turnover rates carry substantial uncertainty (CV ≈ 17%), the net catabolic rate, knet = kdeg − ksyn, that drives disease progression is well-constrained (CV = 12.5%; Supplementary Table S5). The constraint k_deg_ > k_syn_ was imposed *a priori* based on the requirement that OA cartilage exhibits net matrix loss; relaxing this constraint would allow solutions with k_syn_ > k_deg_ that are biologically implausible for progressive OA. Future studies incorporating direct measurements of cartilage turnover biomarkers (e.g., CTX-II for degradation, CPII for synthesis) could help resolve this correlation by providing independent constraints on synthesis and degradation rates.

## Future directions

Several avenues for model extension and application warrant future investigation. First, integrating MRI-based cartilage compositional biomarkers (dGEMRIC, T2 mapping) could enable more granular calibration of cartilage quality metrics beyond radiographic JSW. Second, incorporating inflammatory biomarkers (CTX-II, COMP, and hs-CRP) as model outputs would permit validation against mechanistic endpoints and the identification of biomarker-defined responder subgroups. Third, expansion to combination therapies, glucosamine plus chondroitin, glucosamine plus anti-inflammatory agents, could inform rational polypharmacy strategies. Fourth, application of the model to support regulatory submissions could exemplify the practical utility of MIDD approaches in the nutraceutical/DMOAD space, potentially contributing to the evidence base for glucosamine’s regulatory status. Fifth, the virtual population framework could be extended to *in silico* clinical trial simulation to optimize study design, sample size, and enrichment strategies for future prospective trials.

The model codebase and parameters have been documented to enable independent replication and extension by the scientific community. As QSP approaches maturity and regulatory acceptance grows under the FDA Modernization Act 2.0 framework, we anticipate more opportunities for computational modeling to accelerate therapeutic development for osteoarthritis and other chronic diseases with long development timelines and high clinical trial attrition rates.

## Conclusion

We developed a mechanistic QSP model for glucosamine sulfate efficacy in knee osteoarthritis that demonstrates close calibration to landmark clinical trials (12% error in treatment effect). External validation against the independent GUIDE 2007 placebo-controlled trial reproduced both arms’ pain trajectories (MAE = 3.8 WOMAC points, all observations within the 90% prediction intervals) but under-predicted the between-arm treatment effect (−1.4 vs. −5.0 points), and the wide prediction intervals limit the strength of this test; placebo-response generalizability remains a noted limitation. Under calibration conditions, virtual population simulations predict that most patients derive benefit (median treatment effect 0.186 mm); however, this prediction is conditional on the model’s assumed anti-catabolic potency. When the model is re-calibrated across the concentration range at which glucosamine’s effects are actually observed *in vitro*, the predicted structural benefit falls below clinically meaningful thresholds at achievable synovial exposures (“Re-calibration across the biologically realistic potency range”). The model’s principal value, therefore, lies not in endorsing glucosamine’s efficacy but in identifying the specific pharmacological conditions, adequate potency, and joint exposure required for a disease-modifying effect, and in showing that current evidence does not establish that these conditions are met.

This work exemplifies the application of Model-Informed Drug Development approaches aligned with the FDA Modernization Act 2.0 vision for advancing computational methodologies in drug development. However, the model’s conclusions are contingent on the calibration data, and recent pharmacokinetic and crystallographic evidence [31, 32] challenges the assumption of formulation-dependent bioavailability differences that has historically been invoked to explain inconsistent clinical results. The framework’s primary value lies in quantifying the sensitivity of clinical outcomes to pharmacokinetic parameters and demonstrating how QSP approaches can structure analysis of complex therapeutic controversies, rather than providing definitive conclusions about glucosamine efficacy. Beyond glucosamine specifically, the present framework illustrates how QSP modeling can support rational development of disease-modifying osteoarthritis therapies by integrating drug exposure, disease biology, and clinical outcomes into a unified predictive structure. Such approaches may reduce late-stage attrition by enabling quantitative evaluation of therapeutic hypotheses prior to large clinical trials. More broadly, mechanistic modeling frameworks that link pharmacology to disease progression may help address longstanding challenges in DMOAD development by improving translational predictability and guiding evidence-based clinical strategies.

## Data Availability

The complete analysis pipeline, including all MATLAB scripts, is publicly available at https://github.com/hamedgilzadkohan/glucosamine-qsp. Model equations are listed in full in the Supplementary Appendix.
