# Supplementary material for: A mechanistic quantitative systems pharmacology framework for glucosamine sulfate in knee osteoarthritis: linking exposure, cartilage biology, and clinical outcomes
Source: J Pharm Pharm Sci. 2026 Oct 9;29:16471. doi: 10.3389/jpps.2026.16471 (PMC13720903; doi:10.3389/jpps.2026.16471)
Supplement: Supplementary file 1 [file DataSheet1.docx]

**6. SUPPLEMENTARY MATERIALS**

**S1. Model Equations and Parameter Details**

**S1.1. Pharmacokinetic Module**

Dynamic one-compartment oral PK model:

$$C_{\mathrm{plasma}}(t) = \frac{F \cdot\mathrm{Dose} \cdot k_{a}}{V_{d} \cdot\left( k_{a} - k_{e} \right)} \cdot\left( e^{-k\_e \cdot t} - e^{-k\_a \cdot t} \right)$$

Synovial fluid concentration:

$$C_{\mathrm{syn}}(t) = R_{syn:plasma} \cdot C_{\mathrm{plasma}}(t)$$

Steady-state synovial concentration (used in model):

$$C_{syn,ss} = R_{syn:plasma} \cdot\frac{F \cdot\mathrm{Dose}}{\mathrm{CL} \cdot\tau}$$

where F_sulfate_ = 0.22, F_HCl_ = 0.11, Vd = 200 L, ka = 0.25 h⁻¹, CL = ke × Vd = 12 L/h, and τ = 24 h. These illustrative parameters yield a terminal half-life of ~12 h; the slightly longer ~15 h reported by Persiani et al. 2005, reflects multi-exponential disposition not captured by this single-compartment approximation. The effective synovial concentration was implemented using a reference-scaling approach: C_eff_ = 0.30 × (F/0.22) × (Dose/1500) µg/mL (see §2.7). All PK parameters were fixed from published population PK studies. The dynamic one-compartment profile in this section is illustrative; the calibrated predictions use the steady-state average synovial concentration (reference-scaling, §2.7) and do not integrate this time-course.

**S1.2. Cartilage Dynamics Module (GAG Turnover)**

GAG turnover with dual-mechanism drug effect (anti-catabolic + anabolic):

$$\frac{d\mathrm{GAG}}{dt} = k_{\mathrm{syn}} \cdot E_{\mathrm{syn}} - k_{\deg} \cdot\left( 1 - \frac{I_{max,deg} \cdot C_{\mathrm{syn}}}{IC_{50,deg} + C_{\mathrm{syn}}} \right) \cdot\mathrm{GAG}$$

where the synthesis stimulation factor is:

$$E_{\mathrm{syn}} = 1 + \frac{E_{max,syn} \cdot C_{\mathrm{syn}}}{EC_{50,syn} + C_{\mathrm{syn}}}$$

with k_syn_ = 0.1753 yr⁻¹, k_deg_ = 0.2073 yr⁻¹, I_max,deg_ = 1.0000, IC_₅₀,deg_ = 3.0 µg/mL, E_max,syn_ = 0.15, EC_₅₀,syn_ = 2.0 µg/mL, and GAG(0) = GAG_₀_ = 0.95 (normalized).

***S1.3. Clinical Outcome Module (JSW and Pain)***

Joint space width is linked to GAG content:

$$\mathrm{JSW}(t) = {JSW}_{0} \cdot\left( \frac{\mathrm{GAG}(t)}{{GAG}_{0}} \right)^{\gamma}$$

where JSW₀ = 4.2 mm (baseline medial tibiofemoral JSW) and γ = 1.0 (linear mapping).

Pain is modeled as a composite of baseline severity, structural deterioration from cartilage loss, placebo response, and direct drug effect:

$$\mathrm{Pain}(t) = {Pain}_{0} + \alpha_{\mathrm{struct}} \cdot\max\left( 0, {GAG}_{0} - \mathrm{GAG}(t) \right) - P_{\max} \cdot\left( 1 - e^{-k\_pl \cdot t} \right) - \frac{\beta_{\mathrm{drug}} \cdot C_{\mathrm{syn}}}{EC_{50,pain} + C_{\mathrm{syn}}}$$

where Pain₀ = 46.65 (baseline WOMAC pain, 0–100 scale), α_struct = 150 WOMAC points per unit GAG (pain sensitivity to cartilage loss), β_drug_ = 5.0 WOMAC points (maximum direct analgesic effect), EC_₅₀,pain_ = 3.0 µg/mL, P_max_ = 17.62 WOMAC points (maximum placebo response), and k_pl_ = 0.02 day⁻¹ (placebo onset rate).

**S2. Supplementary Tables**

**Table S1.** Prior distributions and bounds for the five calibrated parameters. All other model parameters were fixed from the literature.

| **Parameter** | **Description** | **Lower** | **Upper** | **Initial** | **Prior Distribution** |
| --- | --- | --- | --- | --- | --- |
| k_syn_ | GAG synthesis rate (yr⁻¹) | 0.01 | 0.20 | 0.10 | Uniform [0.01, 0.20] |
| k_deg_ | GAG degradation rate (yr⁻¹) | 0.01 | 0.25 | 0.12 | Uniform [0.01, 0.25] |
| I_max,deg_ | Max inhibition of degradation | 0.1 | 1.0 | 0.80 | Uniform [0.1, 1.0] |
| Pain_0_ | Baseline WOMAC pain | 35 | 55 | 45 | Uniform [35, 55] |
| P_max_ | Max placebo response (WOMAC) | 10 | 30 | 15 | Uniform [10, 30] |

**Table S2.** Bootstrap parameter estimates (N = 1000 replicates, 999 valid). Estimate = calibrated value from base model. Boot Mean and Boot Median = mean and median of valid replicates. CV = coefficient of variation. Percentiles define the 95% nonparametric confidence interval.

| **Parameter** | **Estimate** | **Boot Mean** | **Boot Median** | **CV (%)** | **2.5th %ile** | **97.5th %ile** | **Notes** |
| --- | --- | --- | --- | --- | --- | --- | --- |
| k_syn_ | 0.1753 | 0.1824 | 0.2000 | 17.3 | 0.0932 | 0.2000 | r(k_deg_) = 0.993 |
| k_deg_ | 0.2073 | 0.2140 | 0.2314 | 16.7 | 0.1127 | 0.2402 | r(k_syn_) = 0.993 |
| I_max,deg_ | 1.0000 | 0.9814 | 1.0000 | 9.0 | 0.6348 | 1.0000 | 94.6% at bound |
| Pain_0_ | 46.6519 | 45.1893 | 45.7021 | 3.8 | 40.4893 | 46.9651 | r(P_max_) = 0.965 |
| P_max_ | 17.6245 | 19.6969 | 20.2752 | 11.4 | 13.5841 | 22.3697 | r(Pain_0_) = 0.965 |

**Note:** *k_syn_ and k_deg bootstrap medians pile up at the upper bound due to the enforced constraint k_deg_ > k_syn_ and the high correlation (r = 0.993). I_max,deg_ shows 94.6% of replicates at the physiological upper bound (≥0.95). Pain_0_ and P_max_ are correlated (r = 0.965), reflecting the trade-off between baseline pain and placebo response magnitude.*

**Table S3.** Bootstrap parameter correlation matrix (N = 999 valid replicates). Values in bold red indicate |r| > 0.9.

|  | **k_syn_** | **k_deg_** | **I_max,deg_** | **Pain_0_** | **P_max_** |
| --- | --- | --- | --- | --- | --- |
| **k_syn_** | 1.000 | 0.993 | −0.015 | −0.025 | 0.014 |
| **k_deg_** | 0.993 | 1.000 | 0.044 | −0.020 | 0.028 |
| **I_max,deg_** | −0.015 | 0.044 | 1.000 | 0.048 | 0.081 |
| **Pain_0_** | −0.025 | −0.020 | 0.048 | 1.000 | 0.965 |
| **P_max_** | 0.014 | 0.028 | 0.081 | 0.965 | 1.000 |

**Note:** *k_syn_ and k_deg_ are highly correlated (r = 0.993), indicating only their net difference (k_net_ = k_deg_ − k_syn_) is independently identifiable. Pain_0_ and P_max_ are also strongly correlated (r = 0.965), reflecting the inherent trade-off between baseline pain level and placebo response.*

**Table S4.** Local sensitivity analysis (±20% perturbation)

Base case outputs: treatment effect = 0.211 mm; placebo JSW change = −0.214 mm; Week 24 placebo pain = 31.1. Sensitivity Index (SI): High (>20% change in output), Moderate (5–20%), Low (<5%).

| **Parameter** | **Base Value** | **Perturbation** | **Affected** | **SI (JSW)** | **SI (Pain)** |
| --- | --- | --- | --- | --- | --- |
| JSW_0_ | 4.2 mm | ±20% | All endpoints | High | Low |
| F_sulfate_ | 0.22 | ±20% | JSW + Pain | Moderate | Moderate |
| IC_50,deg_ | 3.0 µg/mL | ±20% | JSW | Low | Low |
| k_syn_ | 0.175 year⁻¹ | ±20% | JSW | Moderate | Low |
| E_max,syn_ | 0.15 | ±20% | JSW | Low | Low |
| EC_50,syn_ | 2.0 µg/mL | ±20% | JSW | Low | Low |
| GAG_0_ | 0.95 | ±20% | JSW | Moderate | Low |
| k_deg_ | 0.207 year⁻¹ | ±20% | JSW | Moderate | Low |
| I_max,deg_ | 1.000 | ±20% | JSW + Pain | High | Moderate |
| β_drug_ | 5.0 | ±20% | Pain | Low | Moderate |
| α_struct_ | 150 | ±20% | Pain | Low | Moderate |
| k_pl_ | 0.02 day⁻¹ | ±20% | Pain | Low | Moderate |
| P_max_ | 17.62 | ±20% | Pain | Low | High |
| Pain_0_ | 46.65 | ±20% | Pain | Low | High |
| F_HCl_ | 0.11 | ±20% | JSW + Pain | Moderate | Moderate |
| EC_50,pain_ | 3.0 µg/mL | ±20% | Pain | Low | Low |

**Table S5.** Derived parameters computed from bootstrap distributions. k_net represents the identifiable quantity governing the rate of cartilage loss.

| **Parameter** | **Definition** | **Value** | **95% CI** | **CV (%)** | **Interpretation** |
| --- | --- | --- | --- | --- | --- |
| k_net_ | k_deg_ − k_syn_ | 0.032 yr⁻¹ | [0.024, 0.040] | 12.5 | Net catabolic rate |
| k_deg_ / k_syn_ | Degradation/synthesis ratio | 1.18 | — | — | 18% excess degradation |
| t½,net | ln(2) / k_net_ | ~22 yr | — | — | Net GAG half-life (OA) |

**Table S6.** Parameter bounds and biological constraints are enforced during calibration and bootstrap resampling.

| **Constraint** | **Type** | **Value** | **Source** | **Rationale** |
| --- | --- | --- | --- | --- |
| k_deg_ > k_syn_ | Structural | Enforced | Biological | OA requires net cartilage degradation |
| 0 < I_max,deg_ ≤ 1 | Bound | [0.10, 1.00] | Pharmacological | Fractional inhibition cannot exceed 100% |
| GAG_0_ = 0.95 | Fixed | 0.95 | Assumed | Normalized GAG content for mild OA |
| Pain_0_ ∈ [35, 55] | Clinical | WOMAC scale | Literature | Typical moderate OA baseline |
| P_max_ > 0 | Positivity | [10, 30] | Literature | Placebo response is positive |

**Supplementary Table S7.** Risk of Bias Assessment for Core Calibration and Validation Studies (Cochrane RoB 2)

| **Study** | **Randomization Process** | **Deviations from Intervention** | **Missing Outcome Data** | **Measurement of Outcome** | **Selection of Reported Result** | **Overall Risk** |
| --- | --- | --- | --- | --- | --- | --- |
| Reginster 2001 | Low | Low | Low | Low | Low | Low |
| Pavelka 2002 | Low | Low | Low | Low | Low | Low |
| GAIT 2006 | Low | Low | Some concerns^†^ | Low | Low | Some concerns |
| GUIDE 2007 | Low | Low | Low | Low | Low | Low |
| MOVES 2015 | Low | Low | Low | Some concerns^‡^ | Low | Some concerns |

*Risk of bias was assessed using the Cochrane Risk of Bias 2 (RoB 2) tool for randomized trials.*

*^†^GAIT 2006: "Some concerns" for missing outcome data due to differential dropout rates between arms (glucosamine: 21% vs. placebo: 18%) and the unusually high placebo response rate (60%) that may reflect population selection bias rather than true treatment effect dilution.*

*^‡^MOVES 2015: "Some concerns" for measurement of outcome because outcome assessors were not blinded to treatment allocation in this open-label non-inferiority trial, potentially introducing measurement bias favoring the combination therapy arm.*

***Abbreviations****: GAIT = Glucosamine/Chondroitin Arthritis Intervention Trial; GUIDE = Glucosamine Unum In Die Efficacy; MOVES = Multicentre Osteoarthritis interVEntion trial with Sysadoa.*

**Supplementary Table S8.** Model re-calibration across a wide range of assumed anti-catabolic potency (IC_50,deg_). All five calibration parameters were re-estimated at each IC_50,deg_ value using the same objective function and bounds as the primary calibration. Inhibition values are computed at the model reference concentration (0.30 µg/mL) and at the highest measured steady-state synovial concentration (0.78 µg/mL; 4.34 µM, Persiani 2007).

| **IC_50,deg_ (µg/mL)** | **k_syn_ (yr⁻¹)** | **k_deg_ (yr⁻¹)** | **I_max,deg_** | **SSE** | **3-yr JSW TE (mm)** | **Responders (%)** | **Inhib. @0.30 µg/mL** | **Inhib. @0.78 µg/mL** |
| --- | --- | --- | --- | --- | --- | --- | --- | --- |
| **3.0** | **0.200** | **0.235** | **1.000*** | **5.74** | **0.231** | **98** | **9.1%** | **20.6%** |
| 10 | 0.200 | 0.227 | 1.000* | 9.93 | 0.098 | 36 | 2.9% | 7.2% |
| 30 | 0.200 | 0.225 | 1.000* | 12.44 | 0.058 | 0 | 1.0% | 2.5% |
| 100 | 0.200 | 0.224 | 1.000* | 13.47 | 0.044 | 0 | 0.3% | 0.8% |
| 300 | 0.200 | 0.224 | 1.000* | 13.78 | 0.040 | 0 | 0.1% | 0.3% |
| 1000 | 0.200 | 0.224 | 1.000* | 13.89 | 0.038 | 0 | 0.03% | 0.1% |

** I_max,deg_ estimate pinned at its upper bound (1.0) at every IC_50,deg_ value, indicating the model cannot compensate for reduced potency. The re-calibration freely re-estimated all five parameters at each IC50,deg. At 3.0 µg/mL this yielded SSE = 5.74 with ksyn at its upper bound (0.200), marginally below the primary calibration's SSE of 5.99; the primary fit retained an interior ksyn (0.175) and was not re-optimized under the wider bounds used here. Higher SSE at larger IC50,deg indicates progressively worse agreement with calibration data.*

**S3. Supplementary Figures**

**Figure S1.** Profile likelihood analysis for parameter identifiability. Each panel shows the change in objective function (ΔSSE) as a single parameter is varied while re-optimizing all others. Orange dashed line = χ²(1)/2 = 1.92 threshold defining the 95% confidence interval. Green vertical line = calibrated estimate. Gray dotted lines = 95% CI bounds. Three parameters (k_syn, Pain0, Pmax) show defined minima, confirming practical identifiability; k_deg and Imax,deg do not. I_max,deg_ does not cross the threshold, indicating its CI extends to the upper bound (1.0). k_deg_ approaches but does not cross the threshold at the upper range.

**Figure S2.** Residual diagnostics for model calibration. (A) JSW residuals vs. predicted values for placebo (gray) and treatment (green) arms; dashed curve shows loess trend. (B) JSW residuals vs. time, showing no systematic temporal drift. (C) WOMAC pain residuals vs. predicted pain scores. (D) Weighted residuals distribution for JSW (blue) and WOMAC (orange) with standard normal overlay (dotted curve). One-sample t-test on weighted residuals: t = −0.947, p = 0.357 (N = 17 residuals; one baseline target excluded because Pain₀ is a free parameter), indicating no systematic bias.

**Figure S3.** Bootstrap parameter distributions (N = 999 valid replicates out of 1000). Each panel shows the marginal distribution (histogram with kernel density overlay) for one parameter, with calibrated estimate (green vertical line) and 95% CI bounds (gray dashed lines). The inset text shows the CV and 95% CI for each parameter. Bottom-right panel: parameter correlation heatmap. Notable features: k_syn_–k_deg_ correlation r = 0.993; I_max,deg_ pile-up at upper bound with 94.6% of replicates ≥0.95; Pain_0_–P_max_ correlation r = 0.965.

**Figure S4.** Bioavailability impact on efficacy. Predicted 3-year JSW treatment effect (mm) as a function of oral bioavailability (10–40%). Gray dashed line marks the current crystalline glucosamine sulfate bioavailability (F = 22%). Teal dotted line = minimum clinically important difference (MCID = 0.1 mm). The near-linear relationship suggests that formulation improvements increasing bioavailability could substantially enhance structural modification outcomes.

**Figure S5.** Sensitivity of Week 24 pain score to the placebo rate constant (k_pl_). Pain score plateaus for k_pl_ > ~0.05 day⁻¹ (gray region), indicating the model is insensitive to k_pl_ above this value. The calibrated value (k_pl_ = 0.02 day⁻¹, green region) falls in the sensitive zone where the parameter influences predictions. Gray dashed line = observed GAIT Week 24 placebo pain score (28.6 WOMAC points). This sloppy behavior at high k_pl_ values is consistent with the profile likelihood analysis (Figure S1).

Figure S6. Sensitivity of predicted efficacy to the assumed anti-catabolic potency (IC₅₀_,deg_). Top row (A, B): forward sensitivity analysis with all calibrated parameters held fixed. (A) Achieved degradation inhibition as a function of IC₅₀_,deg_, computed at the highest measured steady-state synovial concentration (0.78 µg/mL; 4.34 µM, Persiani 2007, blue) and at the model reference concentration (0.30 µg/mL, grey). The shaded band marks the approximate concentration range (≈10–1000 µg/mL) over which glucosamine's anti-catabolic effects were observed in vitro (Largo 2003). (B) Predicted 3-year joint space width (JSW) treatment effect as a function of IC₅₀_,deg_; the dashed line indicates the 0.1 mm minimal clinically important difference (MCID). Bottom row (C, D): full re-calibration analysis, in which all five calibration parameters were re-estimated at each IC₅₀,deg value. (C) Calibration goodness of fit (sum of squared errors, SSE); higher values indicate worse agreement with the calibration data. (D) Re-fit maximal degradation inhibition (Iₘₐₓ_,deg_, left axis, blue) and predicted 3-year JSW treatment effect (right axis, orange). Iₘₐₓ_,deg_ remains pinned at its upper bound of 1.0 across the entire range, indicating that the model cannot compensate for reduced potency, while the predicted treatment effect falls below the MCID by IC₅₀_,deg_ ≈ 10 µg/mL, and approaches zero at biologically realistic potency values. Together, these panels show that the model's predicted structural benefit is strongly contingent on the assumed anti-catabolic potency. Numerical values are given in Supplementary Table S8.
