# Supplementary material for: A mechanistic quantitative systems pharmacology framework for glucosamine sulfate in knee osteoarthritis: linking exposure, cartilage biology, and clinical outcomes
Source: J Pharm Pharm Sci. 2026 Oct 9;29:16471. doi: 10.3389/jpps.2026.16471 (PMC13720903; doi:10.3389/jpps.2026.16471)
Supplement: Supplementary file 2 [file Supplementaryfile1.docx]

**A Mechanistic Quantitative Systems Pharmacology Framework for Glucosamine Sulfate in Knee Osteoarthritis: Linking Exposure, Cartilage Biology, and Clinical Outcomes.**

**Marzieh Soheili, Hamed Gilzad Kohan**

**Supplementary Appendix: Model Equations and Parameter Details**

**Pharmacokinetic Module**

Dynamic one-compartment oral PK model:

$$C_{\mathrm{plasma}}(t) = \frac{F \cdot\mathrm{Dose} \cdot k_{a}}{V_{d} \cdot\left( k_{a} - k_{e} \right)} \cdot\left( e^{-k\_e \cdot t} - e^{-k\_a \cdot t} \right)$$

Synovial fluid concentration:

$$C_{\mathrm{syn}}(t) = R_{syn:plasma} \cdot C_{\mathrm{plasma}}(t)$$

Steady-state synovial concentration (used in model):

$$C_{syn,ss} = R_{syn:plasma} \cdot\frac{F \cdot\mathrm{Dose}}{\mathrm{CL} \cdot\tau}$$

where F_sulfate_ = 0.22, F_HCl_ = 0.11, Vd = 200 L, ka = 0.25 h⁻¹, CL = ke × Vd = 12 L/h, and τ = 24 h. These illustrative parameters yield a terminal half-life of ~12 h; the slightly longer ~15 h reported by Persiani et al. 2005, reflects multi-exponential disposition not captured by this single-compartment approximation. The effective synovial concentration was implemented using a reference-scaling approach: C_eff_ = 0.30 × (F/0.22) × (Dose/1500) µg/mL (see Module A: Pharmacokinetics). All PK parameters were fixed from published population PK studies. The dynamic one-compartment profile in this section is illustrative; the calibrated predictions use the steady-state average synovial concentration (reference-scaling, Module A: Pharmacokinetics) and do not integrate this time-course.

**Cartilage Dynamics Module (GAG Turnover)**

GAG turnover with dual-mechanism drug effect (anti-catabolic + anabolic):

$$\frac{d\mathrm{GAG}}{dt} = k_{\mathrm{syn}} \cdot E_{\mathrm{syn}} - k_{\deg} \cdot\left( 1 - \frac{I_{max,deg} \cdot C_{\mathrm{syn}}}{IC_{50,deg} + C_{\mathrm{syn}}} \right) \cdot\mathrm{GAG}$$

where the synthesis stimulation factor is:

$$E_{\mathrm{syn}} = 1 + \frac{E_{max,syn} \cdot C_{\mathrm{syn}}}{EC_{50,syn} + C_{\mathrm{syn}}}$$

with k_syn_ = 0.1753 yr⁻¹, k_deg_ = 0.2073 yr⁻¹, I_max,deg_ = 1.0000, IC_₅₀,deg_ = 3.0 µg/mL, E_max,syn_ = 0.15, EC_₅₀,syn_ = 2.0 µg/mL, and GAG(0) = GAG_₀_ = 0.95 (normalized).

**Clinical Outcome Module (JSW and Pain)**

Joint space width is linked to GAG content:

$$\mathrm{JSW}(t) = {JSW}_{0} \cdot\left( \frac{\mathrm{GAG}(t)}{{GAG}_{0}} \right)^{\gamma}$$

where JSW₀ = 4.2 mm (baseline medial tibiofemoral JSW) and γ = 1.0 (linear mapping).

Pain is modeled as a composite of baseline severity, structural deterioration from cartilage loss, placebo response, and direct drug effect:

$$\mathrm{Pain}(t) = {Pain}_{0} + \alpha_{\mathrm{struct}} \cdot\max\left( 0, {GAG}_{0} - \mathrm{GAG}(t) \right) - P_{\max} \cdot\left( 1 - e^{-k\_pl \cdot t} \right) - \frac{\beta_{\mathrm{drug}} \cdot C_{\mathrm{syn}}}{EC_{50,pain} + C_{\mathrm{syn}}}$$

where Pain₀ = 46.65 (baseline WOMAC pain, 0–100 scale), α_struct = 150 WOMAC points per unit GAG (pain sensitivity to cartilage loss), β_drug_ = 5.0 WOMAC points (maximum direct analgesic effect), EC_₅₀,pain_ = 3.0 µg/mL, P_max_ = 17.62 WOMAC points (maximum placebo response), and k_pl_ = 0.02 day⁻¹ (placebo onset rate).
